# GATA transcription factors coordinate developmental progression in the arabidopsis root

**DOI:** 10.1038/s41467-026-78787-0

**Published:** 2026-11-03

**Authors:** Tom Denyer, Pavel Solansky, Arvid Herrmann, Francesco de Martino, Efthymia Symeonidi, Valentin Brösamle, Marja C. P. Timmermans

**Affiliations:** 1 Center for Plant Molecular Biology, University of Tübingen, Auf der Morgenstelle 32, Tübingen, 72076 Germany; 2 School of Biological Sciences, University of Utah, Salt Lake City, Utah 84112 USA

**Keywords:** Root apical meristem, Plant molecular biology

## Abstract

Proper organ development requires the coordination of cell type-specific differentiation programs with general maturation processes through precise spatiotemporal gene regulation. In plants, where RNA polymerase pausing is not well established, alternative mechanisms must ensure this coordination. Here, using single-cell resolution transcriptomic and chromatin accessibility data, we reconstruct developmental trajectories of the Arabidopsis root ground tissue to investigate these mechanisms. We describe phased transcriptional landscapes governing the transition from stem cell to early differentiation, in which chromatin accessibility marks the activation of key developmental regulators. A major transcriptional switch in the late meristematic zone, underpinned by extensive chromatin remodeling, drives the transition from cell division to differentiation. Leveraging machine learning to examine integrated transcriptomic and chromatin accessibility trajectories, we identify GATA transcription factors (TFs) as central coordinators of this switch; a role we validate experimentally. We show that the GATA TFs sit at the tipping point of a feedback-driven bistable system that integrates inputs from auxin and cytokinin to balance proliferation and differentiation, ensuring temporal precision in root development at both the tissue and organ scales.

## Abstract (web summary)

Cell type-specific differentiation programs rely upon precise gene regulation. Here, using single-cell analysis, the authors show that phased transcriptional landscapes govern the transition from stem cell to early differentiation in Arabidopsis roots and that chromatin accessibility marks the activation of key regulators.

## Introduction

How a single fertilized egg gives rise to a complex multicellular organism, with all its morphological and functionally diverse cell types, is an elemental question in developmental biology. A useful way to visualize the process by which cells and tissues differentiate is through Waddington’s epigenetic landscape^1^. Here, a marble rolling down a hill navigates features of the landscape that, akin to decision making events along a developmental trajectory, guide it through possibilities towards an eventual, normally irreversible, fate at the base of the hill. Aspects of the regulatory landscape controlling various decision points, such as cell fate determining transcription factors (TFs) and signaling components, have been clarified over time^2–5^. However, what is lacking is a comprehensive view of the entire spectrum of gene expression changes that underlies cellular progression. What, for example, induces the necessary cytoskeleton reorganization, polarized cell expansion, and cell type-dependent specializations? Within such a developmental system, cellular differentiation must be tightly balanced with cell proliferation^6^. The correct balance between these processes is critical for determining the overall morphology and size of a developmental structure. As such, their timing must be precisely regulated at the cellular, tissue, and organ-wide scales, the mechanisms of which remain largely unknown. In this regard, high throughput, single cell resolution technologies offer us the opportunity to gain new insights into the diversity of cell types and the paths by which they arise^7–9^. Such concepts have been explored particularly well in some animal systems^10,11^, and experimentation in plants is beginning to exploit these opportunities, as well^12–14^.

The Arabidopsis root presents a powerful system within which to study the dynamics of cell differentiation and its coordination with maturation programs balancing cell proliferation and differentiation. The different cell types of the root are largely organized in concentric rings that originate from a specialized stem cell niche (SCN) near the root tip (Supplementary Fig. 1). The stem cells at the niche divide in a predictable manner, giving rise to daughter cells of distinct identities that undergo several rounds of cell division before visually initiating cell elongation and differentiation programs^15,16^. These processes each occur within clearly defined longitudinal zones of the root, indicating that developmental maturation is indeed precisely regulated across cells of a given tissue and across the root as a whole^15^ (Supplementary Fig. 1). Developmental trajectories of individual cell files are therefore captured along the length of the root, and within these trajectories, general processes of differentiation common to all tissues, occur alongside those limited to specific lineages. A fine example of this has been described in the phloem wherein lineage-specific developmental regulators, the PEAR TFs, interact with those governing general maturation^17^.

Here, to further our understanding of the processes guiding cellular differentiation in the context of general maturation programs of the root, we reconstructed single cell resolution developmental trajectories of the Arabidopsis root ground tissue (GT), comprising the endodermis and cortex, at both transcriptional and chromatin levels. An ideal model, the GT cell files originate from a shared initial (CEI) that undergoes two successive asymmetric divisions, first giving rise to a CEI daughter (CEID), which then generates distinct endodermal and cortex cells that divide anticlinally and specialize as they traverse the length of the root^15^ (Supplementary Fig. 1). We show that cell differentiation in the root manifests from phased expression programs in which chromatin accessibility serves as a strong predictor of developmental gene activation. We further show that a major transcriptomic switch in the late meristem zone (MZ), underpinned by considerable chromatin remodeling, drives the transition from cell division to cell differentiation. Machine learning-based network inference of integrated GT trajectories identifies GATA TFs as core regulators of this transition. We show that these TFs function within a feedback-driven bistable gene regulatory network that integrates auxin- and cytokinin signaling to ensure temporal precision of the proliferation-to-differentiation switch at the cell, tissue, and organ scales.

## Results

### A switch in gene expression profiles from cell proliferation to differentiation

**Fig. 1:**
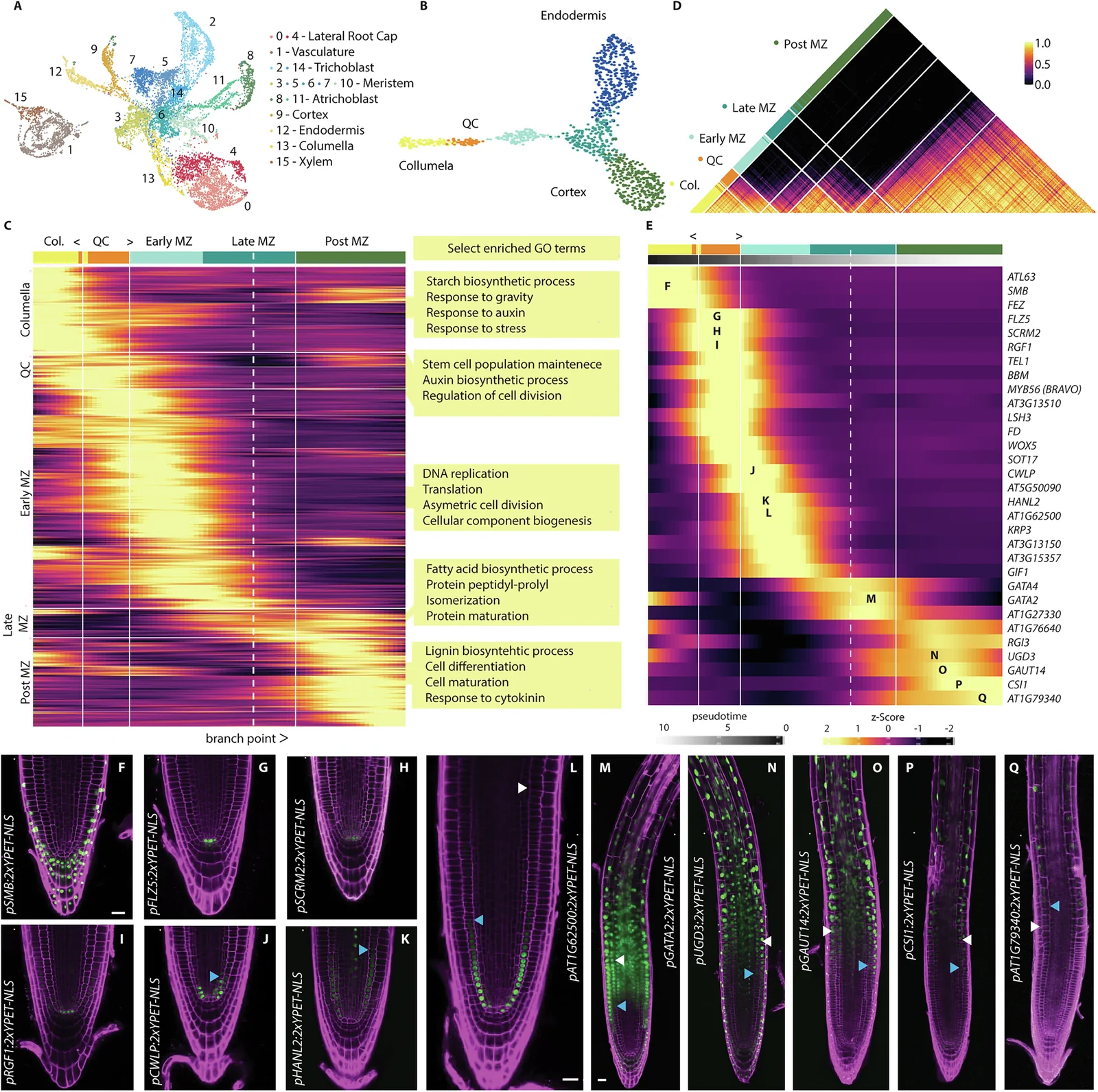
A finely resolved cortex developmental trajectory reveals a switch in gene expression programs within the late meristematic zone (MZ). **A** UMAP of 11,480 scRNA-seq Arabidopsis root profiles resolved as 16 distinct clusters. Differentially expressed genes (DEGs) defining these clusters can be found in Supplementary Data 1. **B** Cells of the columella, QC, and ground tissue (GT, see Supplementary Fig. 2 A for cell selections) form a branched pseudotime-derived developmental trajectory. Cell clusters identified within the trajectory are distinguished by colour. **C** Heatmap of genes changing expression along the cortex-terminal trajectory reveals distinct gene expression phases whose boundaries parallel defined anatomical features discerned based on reporter gene expression (as **E**-**Q**, Supplementary Fig. 2D). Y-axis: genes arranged top to bottom based on maximum expression along pseudotime (x-axis), subsequently categorised into the five anatomical regions. Solid vertical lines: boundaries around the QC and MZ; Dashed vertical line: branch point in the GT trajectory; Top horizontal bar: distribution of cells along pseudotime coloured by cluster assignment. Note the position of the GT branch point in the late MZ. Arrows on either side of the QC indicate bidirectionality of the trajectory from this point. **D** Pearsons’ correlation analysis comparing transcriptomic similarity between cells ordered along pseudotime reveals five distinct gene expression phases (white dotted lines) that correspond with cluster dynamics (B) and anatomical features (**E**-**Q**). **E** Heatmap (as in **C**) showing expression profiles of key marker genes along the cortex-terminal trajectory places the trajectory in anatomical context and reveals its fine resolution. **F**-**Q** Confocal images of transcriptional reporters for select temporal marker genes in the root. Genes shown are highlighted in (**E**). White arrowheads: approximate end of the MZ; Blue arrowheads: latest (**J**–**L**) or earliest (**M**-**Q**) detected expression in the cortex. See also Supplementary Fig. 2D for data on select dual reporters. Scale bars, 20 µm.

To understand the processes guiding cellular differentiation within the root, we used previously described *Arabidopsis thaliana* scRNA-seq data^12,18^ to resolve a high-resolution pseudotemporal developmental trajectory for cells of the GT. From an atlas of ~11,000 cells (Fig. 1A, Supplementary Data 1), those of the SCN, endodermis and cortex were confidently identified based on the expression profiles of established marker genes^12^ (Supplementary Fig. 2A). Reflecting a shared gene expression signature associated with the stem cell state, the SCN cluster includes cells from distinct tissue lineages^12,14,19^. To omit any confounding effects from non-GT cells in the trajectory, cells in the SCN cluster were inspected manually for features distinguishing GT from epidermal- and vasculature-lineages (see methods). This proved to greatly improve the resolution of the final trajectory. Cells with columella identity were included in the trajectory, as this was found to anchor its basal end and to further increase resolution. The final subset of 1,095 cells forms a developmental trajectory that distinctly bifurcates at one end, reflecting the anatomical division of GT cell types within the root (Fig. 1B, Supplementary Fig. 1). A heatmap displaying the expression dynamics of genes varying along the trajectory reveals a seemingly gradual progression of transcriptional changes from the columella/QC end, to a region just before the bifurcation point in the trajectory which is marked by an abrupt switch in gene expression profiles (Supplementary Fig. 2B). These characteristics are even more apparent in individual lineage trajectories, as evidenced by an equivalent expression heatmap of a ‘cortex-terminal’ developmental trajectory (Fig. 1C, Supplementary Fig. 2C, Supplementary Data 1). Analogous to observations of gene expression dynamics along epidermis and phloem trajectories^12,17^, the heatmap reveals five defined phases of expression which respect the trajectory’s cell cluster arrangement (Fig. 1C), as well as the cell-to-cell similarities along pseudotime defined by Pearson’s correlation analysis (Fig. 1D).

To place the cortex-terminal trajectory in an anatomical context, we generated transcriptional reporter lines to examine the temporal expression patterns of select genes defining the different expression phases (Fig. 1E–Q, Supplementary Fig. 2D, E). Conversely, we also examined the expression profiles of previously characterised marker genes along the trajectory^12,20^ (Supplementary Data 5). As such, boundaries between the columella and QC, and between the QC and cortex/endodermal initial cell (CEI) could be accurately pinpointed to either side of the second expression phase of the trajectory (Fig. 1C). For example, *FLZ5*, *SCREAM2* (*SCRM2)*, and *RGF1*, which along with the known markers, *WOX5* and *MYB56 (BRAVO)*, mark the second phase, show QC-specific expression profiles (Fig. 1E, G–I, extended gene names and references can be found in Supplementary Data 5). Bordering the boundaries of QC expression, expression of *FEZ* marks the adjacent columella initial cells, whereas genes such as *CWLP* and *AT1G62500* initiate expression in the CEI^12,21^ (Fig. 1E, F, J, Supplementary Fig. 2D). Expression of *AT1G62500*, *HANL2*, and *GIF1*, which define the third expression phase (Fig. 1E, K, L), extends typically 12-15 cells from the CEI and encompasses the rapidly dividing transit-amplifying cells of the cortex in the SCN-proximal half of the MZ. The start of the fifth gene expression phase of the trajectory, defined in part by expression of *UGD3, GAUT14, ARABIDOPSIS RESPONSE REGULATOR12* (*ARR12), CSI1* and *AT1G79340*, could likewise be determined to coincide closely with the position where the MZ ends and early differentiation processes begin (Fig. 1C, E, N-Q, Supplementary Fig. 2D). The five phases of gene expression characterizing the cortex-terminal trajectory thus coincide with anatomical features along the longitudinal axis of the root, and correspond to, in order, the columella, QC, early MZ, late MZ, and post MZ. Gene ontology (GO) analysis of the 7,339 DEGs defining the phases revealed characteristics expected for these root zones, further corroborating the annotation (Fig. 1C, Supplementary Data 1).

Strikingly, the switch in gene expression underlying the branch point in the GT trajectory therefore occurs over several cells in the late MZ of the root, well after the defining asymmetric cell divisions at the niche^15^. This characteristic is also observed in the equivalent endodermis-terminal trajectory and is consistent with the temporal expression patterns of genes such as *GATA2, GATA4*, and *AT1G27330*. Expression of these genes, which distinguishes the fourth expression phase of the trajectory, marks the late MZ^20^ (Fig. 1E, M, Supplementary Fig. 2D, Supplementary Data 1). These observations indicate that the cell lineages of the GT remain transcriptomicaly similar well after they have separated physically and with respect to cell fate. The cortex and endodermis thus share a developmental program within the SCN-proximal, early MZ whose expression signature overshadows those of the bespoke cell lineage programs. Indeed, relatively few genes displaying cortex- or endodermis-specificity are prominent in this region^20^. As such, on a transcriptomic level, cell lineage-specific programs only present strongly once cells exit from cell division, accompanied by a dramatic switch in gene expression towards differentiation in cells of the late MZ (Fig. 1C).

### A scATAC-seq atlas to explore gene regulation in the Arabidopsis root

**Fig. 2:**
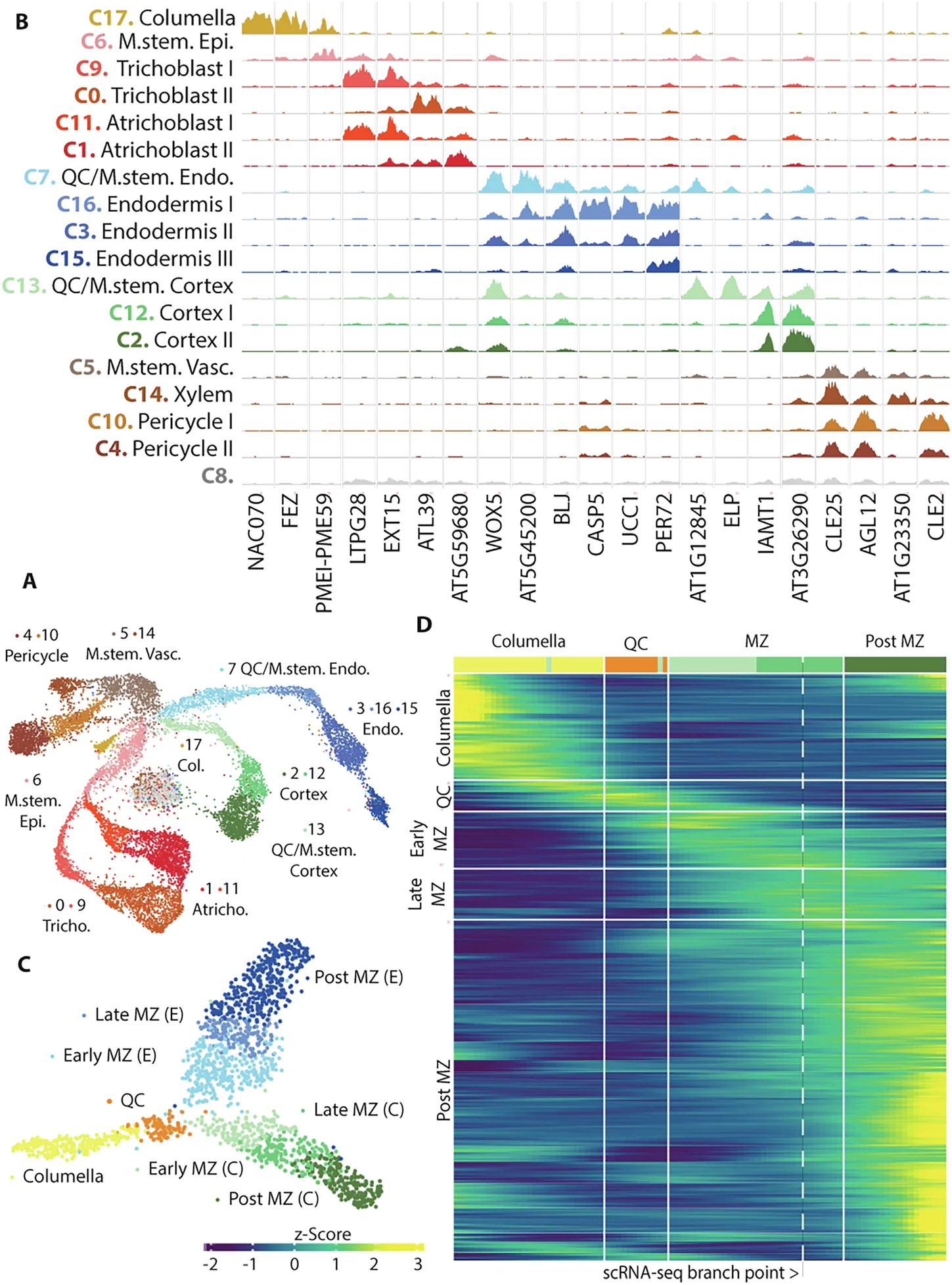
Chromatin accessibility analysis of Arabidopsis root nuclei identifies regulatory dynamics underlying GT development. **A** An atlas of 14,466 Arabidopsis root nuclei assayed using scATAC-seq is presented as 18 clusters by UMAP. Differentially accessible regions (DARs) defining these clusters and annotation of the atlas can be found in (**B**) and Supplementary Data 2. M.stem. – Meristematic, Vasc. – Vasculature, Col. – Columella, Endo. – Endodermis, Tricho. – Trichoblast, Atricho. – Atrichoblast, Epi. – Epidermis. **B** Cluster-aggregated chromatin accessibility near known marker genes (columns) illustrating DARs defining distinct clusters (rows). Each column shows a region of 500–1000 bp positioned within 2,5 kb of the gene body in either direction. **C** 1734 nuclei from the columella, QC, cortex and endodermis form a branched pseudotime-derived developmental trajectory. Cell clusters identified within the trajectory are distinguished by colour and annotated through label transfer from the equivalent transcriptomic progression. C - cortex; E - endodermis. Note, the position of the GT branch point at the early MZ. **D** Chromatin accessibility heatmap for genomic regions that change accessibility along the cortex-terminal trajectory, showing the marked change in chromatin accessibility at the onset of differentiation. Solid vertical lines: boundaries around the QC and MZ; Dashed vertical line: approximate point at which the scRNA-seq GT trajectory bifurcates; Top horizontal bar: distribution of nuclei along pseudotime coloured by cluster assignment.

To understand how the phases of gene expression manifest, and particularly how the switch in gene expression programs in the late MZ is coordinated, we applied chromatin accessibility profiling to explore corresponding dynamic changes in the chromatin landscape. In combination with data on TF binding motifs and gene expression, chromatin accessibility is particularly informative for predicting gene regulatory events that underpin developmental progressions^22–24^. Using a single-cell resolution sequencing assay for transposase-accessible chromatin (scATAC-seq), we generated a complementary atlas of accessible chromatin regions (ACRs). In total, 41,350 non-redundant ACRs (median 526 bp width) were identified across 14,466 Arabidopsis seedling root nuclei (Fig. 2A, median 14,659 unique fragments per nucleus). Indicative of the quality of the scATAC-seq atlas, chromatin accessibility in the pseudo-bulked scATAC-seq dataset is highly correlated to bulk ATAC-seq data of root tips^24^ (R = 0.91; Supplementary Fig. 3A). In addition, the majority of ACRs are located around transcription start sites (TSS), or within 1 kb upstream of a TSS (Supplementary Fig. 3B), in line with previous observations^24,25^.

Nuclei within the atlas form 18 clusters (Fig. 2A). These are distinguished by 18,001 non-redundant differently accessible (DA) chromatin regions (q < 0.05, Log2FC > 1), suggesting that ~40% of ACRs contain information contributing to the identity, state, or differentiation of cells within the Arabidopsis seedling root (Supplementary Data 2). The clusters were annotated based on collated gene activity scores (Fig. 2A, B, Supplementary Data 2), which revealed that the arrangement of nuclei within the scATAC-seq atlas recapitulates the organization of the root both with respect to the spatial arrangement of cell types and their state of differentiation (Fig. 2A, B, Supplementary Fig. 1). Nuclei from cells of the SCN form a central core in the UMAP from which cells of different identities radiate in contrasting directions based on lineage and differentiation state. Specifically, the spatial arrangement of cell branches for the vasculature, endodermis, cortex, and epidermis in the atlas mirrors that of the actual root (Fig. 2A). A similar spatiotemporal organization is also apparent in scRNA-seq atlases^12,14^, suggesting that chromatin accessibility landscapes are closely coupled with transcriptional programs characterizing distinct cell types and states in the root.

### DA chromatin underpins expression of key developmental regulators

To examine how chromatin accessibility changes as GT development progresses, we constructed a pseudotemporal trajectory based on the 1,746 nuclei in the atlas corresponding to the cortex, endodermis, QC and columella^18^ (Supplementary Fig. 3C, see methods). As with its scRNA-seq equivalent, this revealed a trajectory with columella cells at one end and separate cortex and endodermal branches at the other (Fig. 2C). Annotation of the scATAC-seq trajectory through label transfer from the equivalent transcriptomic progression pinpointed the boundaries around the QC and the MZ regions (Supplementary Fig. 3D). This annotation was affirmed with an independent multi-omics integration tool, GLUE^26,27^ and is supported by cluster-aggregated chromatin accessibility near marker genes (Supplementary Fig. 3E, F, Supplementary Data 3). Strikingly, contrary to the transcriptomic profile, cortex and endodermal fates distinguish at the chromatin level shortly after the QC in the early MZ, likely closely following the specification of CEIs at the QC, in-line with the origin of the two GT lineages via asymmetric division of the CEI daughter. The considerable number of dynamically accessible chromatin regions (DARs) between cortex and endodermis in the early MZ (278) corroborates the early chromatin-level bifurcation (Supplementary Fig. 3D, Supplementary Data 3). One explanation for the disparity between the two trajectories is that the common gene expression program in the early MZ proceeds without major changes in chromatin accessibility. Support for this comes from analysis of the cortex-terminal trajectory, wherein the early MZ is distinguished by comparatively few DARs (Figs. 1C-D, 2D, Supplementary Data 3). Contrary to the large number of region-specific DEGs (4,183), only 366 DARs are detected in the cortex cells of the early MZ. Accordingly, transcriptional changes in these cells occur largely within stable chromatin states, which given the prominence of cell division-related gene expression, are most likely inherited from embryogenesis. An alternative hypothesis is that lineage-specific chromatin accessibility emerges before overt transcriptional differentiation through priming^28^, but this idea is not supported currently.

**Fig. 3:**
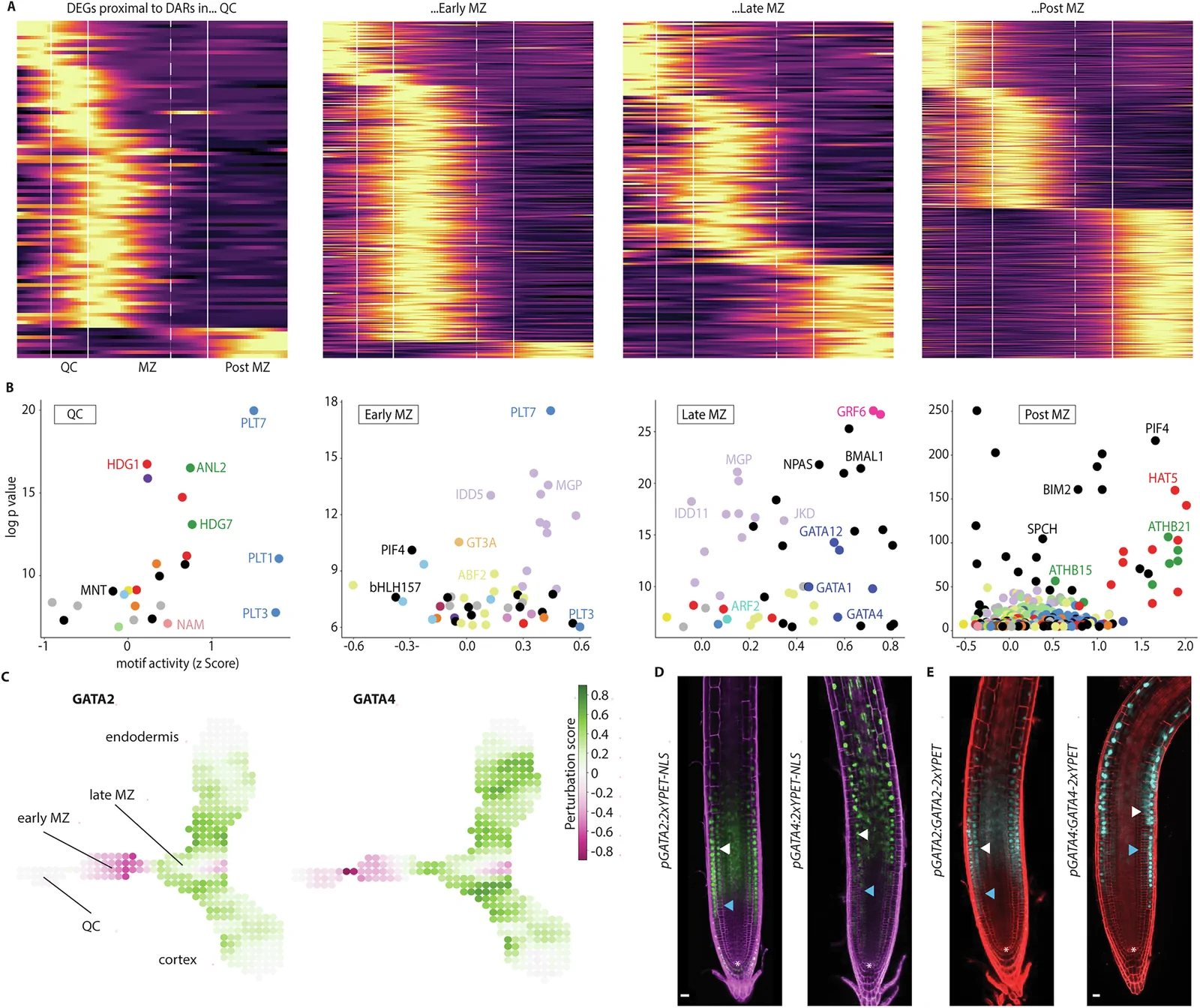
Defined transcription factors coordinate the phased gene expression program underlying root zonation. **A** Expression heatmaps of cortex-terminal trajectory DEGs that are positioned within 2.5 kb of region-specific DARs in the equivalent scATAC-seq trajectory showing increased accessibility (L-R: QC, early MZ, late MZ, and post MZ). **B** Scatter plots showing TF binding motifs significantly enriched under DARs marking each zone of the root. Select motifs are highlighted. Colors indicate DNA binding motifs belonging to TF (sub)families as defined in JASPAR (see Supplementary Data 3 for further details). **C** Perturbation scores (PS) from CellOracle simulations of GATA2 and GATA4 loss-of-function projected onto the GT developmental trajectory show negative values, indicating inhibition of progression, in the early and late MZ, suggesting impaired differentiation and accumulation of undifferentiated meristematic cells upon TF loss. **D** Confocal images of promoter-reporter fusions for *GATA2* and *GATA4* show expression of both genes initiates in the late MZ, with the exact start of gene expression varying slightly between tissue layers. **E** Translational reporter fusions show GATA2 and GATA4 accumulation in cells of the late MZ extending variably into the post MZ. Blue arrowheads: first detected expression in the cortex; White arrowheads; approximate end of the MZ. Asterixis; QC. Scale bar, 20 µm.

Of note, a high number of early MZ DARs ( ~ 83%) are positioned within 2.5 kb of a gene showing differential expression along the cortex trajectory (Supplementary Data 1, 3). Expression of those DEGs is primarily associated with the early MZ (Fig. 3A). Similarly, DARs marking the QC are overwhelmingly associated with DEGs that are active in the QC and early MZ (Fig. 3A). This indicates that the DARs characterizing the niche and transit amplifying cells of the root primarily promote the activation of gene expression. Markedly, many of the genes regulated at the chromatin level in these regions encode TFs and other key developmental regulators. For example, genes for the QC-specifying TFs PLETHORA1 (PLT1) and BABYBOOM (BBM), the AUXIN RESPONSE FACTORS, ARF6 and ARF16, the IAAs, BODENLOS (BDL) and SOLITARY ROOT (SLR), the PIN3 auxin efflux carrier, and the cell proliferation mediators RGF8, PERIANTHIA (PAN), MAP65-3, CYCLIN B2;3, and SMO4 are proximal to chromatin regions DA in the QC or early MZ (Supplementary Data 3, 5). As such, regions of DA chromatin in cells of the root SCN and adjacent transit-amplifying cells are overwhelmingly associated with the transcriptional activation of key developmental regulators in these cells. Only a few of the DARs identified in these root zones are linked to genes involved in differentiation, perhaps arguing against the concept of transcriptional priming^28^ (Fig. 3A).

### The late meristem zone as a hub of regulatory rewiring

The relationship between chromatin accessibility and gene expression in post-MZ cells appears different from that observed in the early MZ. Many more DARs (2728) are detected in the post-MZ cortex cells, indicative of considerable chromatin reorganization associated with cell elongation and differentiation, but fewer DARs are near genes dynamically expressed along the cortex trajectory. Scrutiny of additional transcriptomic datasets^14,20^, however, indicates nearly half of regulation stemming from these DARs manifests in expression in cells further up the root, beyond those captured in the scRNA-seq trajectory. Like the genes regulated by DA chromatin in the early MZ, many genes proximal to post MZ DARs have recognized developmental roles in the root. These include a range of cell elongation and differentiation factors, including *ARR10*, *WOODEN LEG* (*WOL*), *UPB1*, *CSI1* and *BIM1* (Supplementary Data 3, 5). However, contrary to observations in the early MZ, around a third of DEGs proximal to post MZ DARs are expressed preferentially in the QC and early MZ, such as *WOX5* and *TMO7* (Fig. 3A, Supplementary Data 3, 5). These DARs identify a subset of genes with functions early in the cortex trajectory that are actively repressed once cells start to differentiate. This includes genes which are also proximal to early MZ - or QC DARs, pointing to active, dual-regulation of time expression along the developmental trajectory. Importantly, early-expressing genes proximal to post MZ DARs account for only 9% of genes downregulated upon differentiation, indicating again that much of the gene expression related to cell division in the early MZ is not regulated at the chromatin accessibility level.

Notably, while few dynamically expressed genes mark the cells of the late MZ, 323 DARs are detected in these cells, a number comparable to that in the early MZ. Over half of the DARs are associated with genes showing dynamic expression along the cortex trajectory, with a near equal number expressed before (QC, early MZ) and after (post MZ) the late MZ. Among the former are *WOX5*, *PAN*, and other meristem regulators, while genes expressed in the post-MZ are linked to differentiation-specific processes such as cell wall loosening and endoreduplication (Supplementary Data 3, 5). These characteristics suggest that cells of the late MZ host regulatory machinery required for the transition from cell proliferation to differentiation. Although chromatin regulation continues into the post MZ, the switch in gene expression evident at the transcriptomic level in the late MZ (Fig. 1C), pinpoints this region as a regulatory ‘hub’, coordinating the repression of core genes underlying SCN- and cell proliferation-related processes with the timed expression of key regulators of cell elongation and differentiation.

### GATA TFs characterize the regulatory hub in the late meristem zone

To arrive at candidate TFs coordinating the phased gene expression structure of the GT trajectory, we examined the enrichment of known TF binding motifs within region-specific DARs^29^ (Fig. 3B, Supplementary Data 4). A PLT motif is strongest enriched in DARs defining the QC and early MZ, where these TFs serve to promote stem cell function and cell division, respectively^30,31^ (Figs. 1C, 3B). In addition, an abundance of predicted PLT2 targets^32^ are proximal to DA chromatin in the QC and/or early MZ, with activated targets expressed early and repressed targets late in the cortex-terminal transcriptomic trajectory (Supplementary Data 3). Further supporting the notion of PLT TFs dictating the early transcription phases of the cortex trajectory, DNA-binding motifs for HOMEODOMAIN GLABROUS (HDG) TFs, interaction partners of PLT in controlling cell proliferation^33^, are also highly enriched within QC DARs. Contrary to the QC, DARs in the early- and late MZ show a strong enrichment of IDD family binding motifs. These are shared by the BIRD family of C2H2 zinc-finger TFs, members of which are expressed in a corresponding pattern in the root (Supplementary Fig. 3G). The prominence of binding motifs for these TFs also matches their key roles in mediating SHR/SCR positioning and coordinating the formative divisions characterizing GT development^34^. Prominently associated with post-MZ DARs are binding motifs for members of the bHLH and BES1 families, which cooperate to guide BR responses integral to cell elongation^35,36^ (Fig. 3B, Supplementary Fig. 3G, Supplementary Data 4). The patterns of motif enrichment for these TFs along the chromatin trajectory thus mirror expectations, pointing again to the quality and depth of the data. Accordingly, clear predictions about new regulators that coordinate expression changes along the GT trajectory emerge. For example, several MYB, Homeobox, and HD-ZIP DNA-binding motifs are highly enriched in the post-MZ DARs (Fig. 3B, Supplementary Data 4), pointing to prominent contributions from these TF families to cellular differentiation in the root.

Within the late MZ DARs, binding motifs for GROWTH REGULATORY FACTORS (GRFs) stand out as a prominent, region-specific set (Fig. 3B, Supplementary Data 3, 5). Although *GRF* transcript levels are highest in the transit-amplifying cells of the early MZ (Supplementary Fig. 3G), protein persistence, as reported for the PLT TFs^30^, may extend their activity into the late MZ. GRFs are known repressors of *PLT* expression^37^, in line with enrichment of their binding motifs in the late MZ DARs. However, given this enrichment, GRFs likely have a broader influence on gene expression along the cortex-terminal trajectory, potentially contributing to the gene expression switch characterizing this region.

To gain further mechanistic insight into this gene expression switch, we applied CellOracle^38^ to the integrated transcriptomic and chromatin accessibility GT trajectory. This machine learning-based framework infers spatiotemporally resolved gene regulatory interactions specifically to simulate effects on cell progression following TF perturbation, providing a systematic and intuitive interpretation of context-dependent TF function in regulating cell identity. To assess the predictive validity of the inferred interactions, we examined network centrality measures, which reflect the regulatory importance of each node across the trajectory. PLT family members ranked highest in the QC cluster, alongside SCHIZORIZA (SCZ), a TF involved in the asymmetric division of stem cells^39^ (Supplementary Data 4). Meanwhile, MAGPIE, one of the BIRD TFs, emerged as a major hub in both the early and late MZ, and ARR1 and ARR12 are prominent in the post MZ, in line with their established developmental roles^16,34,40^ (Supplementary Fig. 3H, Supplementary Data 4). The recovery of these known regulators at their expected trajectory positions supports the biological fidelity of the inferred network and points to candidate TFs underlying the late MZ regulatory ‘hub’.

Curiously, despite the prominent enrichment of their motifs in late MZ DARs, the GRFs do not emerge as top network nodes at any position along the GT trajectory. Instead, GATA TFs stand out as prominent nodes in the late MZ (Supplementary Data 4). As with the GRFs, Subfamily 1 (SF1) GATA DNA-binding motifs are uniquely enriched in the late MZ DARs. In addition, *GATA2* and *GATA4*, members of this subfamily, show maximum expression in the late MZ, albeit that expression of *GATA4* commences slightly earlier along the cortex trajectory (Fig. 1E, 1M, Supplementary Fig. 3F-G, Supplementary Data 1, 3). In line with these observations, but notably distinct from the GRFs, GATA2 and GATA4 display particularly high eigenvector centrality specifically within the late MZ, suggesting an important regulatory role in this phase that affects a large number of downstream targets (Supplementary Fig. 3H). Moreover, in silico simulations of loss-of-function perturbations of either TF predict impaired progressions along the GT trajectory, with cells accumulating in the early or late MZ (reflecting the slight distinction in *GATA4* and *GATA2* expression) and failing to transition toward later developmental stages (Fig. 3C). These analyses therefore position the SF1 GATA TFs as potential key regulators in the late MZ promoting the timed progression from cell division to cell differentiation.

### GATA TFs coordinate the transition from cell proliferation to differentiation

To test these in silico predictions, we first established the spatiotemporal context of GATA TF activity in the root using transcriptional and translational reporters for GATA2 and GATA4. Promoter fusions for both genes show expression across tissue layers commencing in the late MZ in close agreement with the expression dynamics inferred from the transcriptomic trajectories (Figs. 1M, 3D, Supplementary Fig. 3G, Supplementary Data 1^41^). Reporter expression extends variably into the post-MZ, potentially reflecting the comparatively long half-life of most proteins relative to RNA. GATA2 and GATA4 protein fusions show similar expression profiles but, particularly in the case of GATA2, dissipates rapidly from the late MZ into the elongating cells of the post MZ (Fig. 3E), thus defining a narrow temporal window consistent with a role in regulating the developmental transition from cell proliferation to differentiation.

In addition, we scrutinized the scATAC-seq dataset to characterize potential direct SF1 GATA TF targets. These are characterized by the presence of accessible chromatin within the promoter region (up to 1 kb upstream of the TSS) containing a SF1 GATA DNA-binding motif^29^. In the absence of a predicted GATA2 motif, we used the highly-conserved DNA-binding motifs of GATA4 and GATA12 as representatives for the subfamily (Supplementary Fig. 4A). Given the high conservation of binding motifs within plant TF subfamilies^42,43^, and the near identity of the GATA4 and GATA12 motifs, we expect results based on these motifs to be representative of GATA2 as well. Of the 41,350 ACRs identified across the atlas, 3,941 contain at least one of the GATA motifs, and these map to the promoters of 3,799 genes (Supplementary Data 4). A further 1,009 genes, predicted to be GATA4 targets by DAP-seq^44^, were added to this target set, to form a comprehensive list of predicted SF1 GATA targets (Supplementary Data 4).

**Fig. 4:**
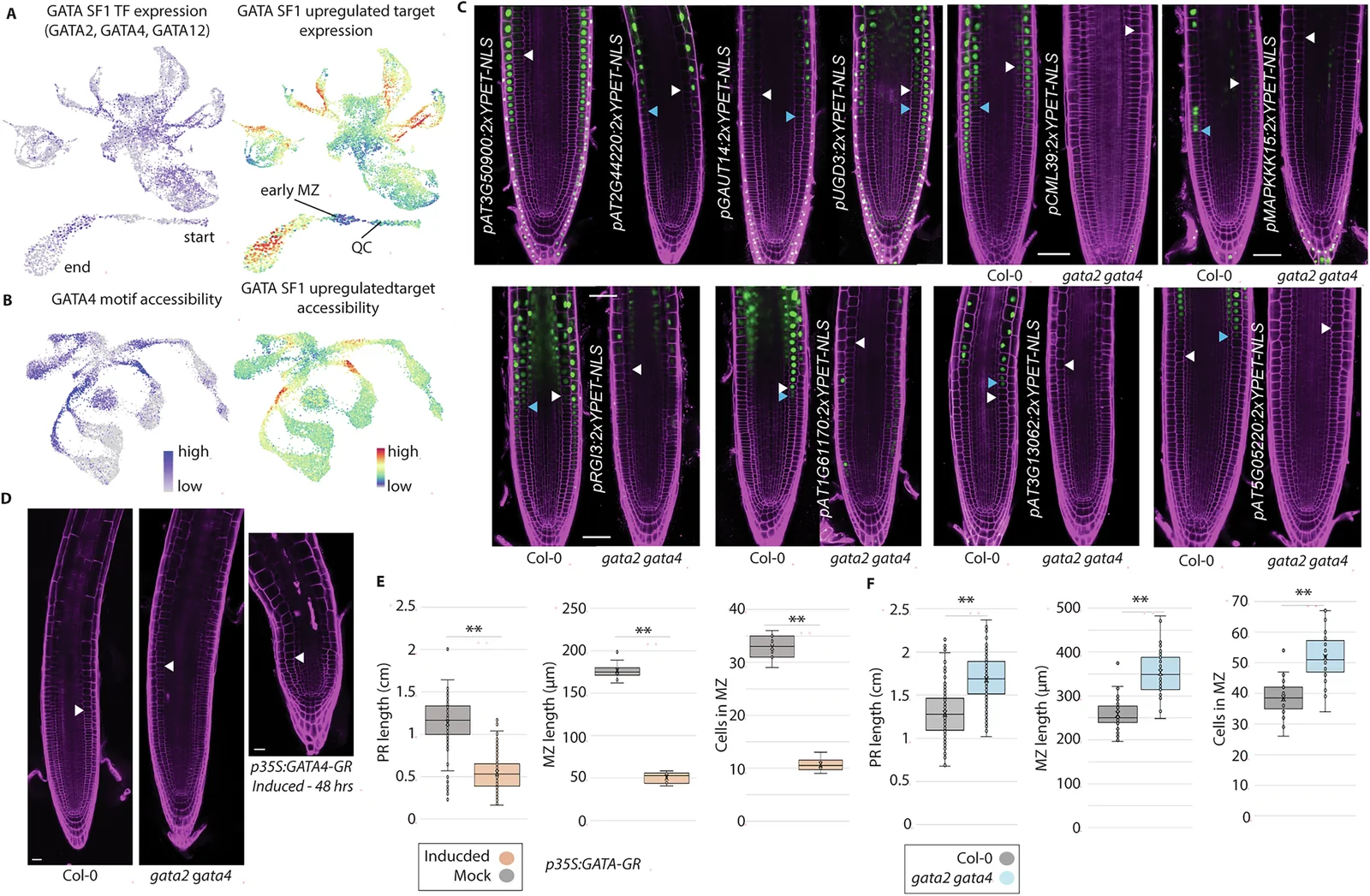
SF1 GATA TFs determine root meristem size through activation of differentiation genes. **A** UMAPs showing enrichment of expression (module score) for the SF1 GATA TFs (left) and their predicted targets upregulated upon transient GATA2 or GATA4 expression (Supplementary Data 4, right) in the scRNA-seq atlas (above) and in the cortex-terminal trajectory (below). **(B)** Left: Motif activity score illustrating accessibility of the GATA4 motif within the scATAC-Seq atlas. The GATA12 motif shows a comparable profile (Supplementary Fig. 4C). Right: Enrichment of chromatin accessibility (module score) in promoters of predicted SF1 GATA targets upregulated upon transient GATA2 or GATA4 expression (Supplementary Data 4). **C** Confocal images of transcriptional reporter fusions for predicted SF1 GATA targets show expression across different cell layers generally initiating around the late MZ. Expression of tested targets is reduced in *gata2 gata4* compared to Col-0. **D** Confocal images of 5-day-old roots show dramatic changes in meristem size upon perturbation of SF1 GATA activity (L-R: Col-0, *gata2 gata4*, ectopic GATA4 overexpression (*p35S:GATA4-GR* – 48 hrs induction with 20 µM dexamethasone)). **E, F** Box plots for primary root (PR) length (left), MZ length (center), and the number of cells in the MZ (right) in 5-day-old roots of *p35S:GATA4-GR* induced (48 hrs) vs mock (**E**), and Col-0 vs *gata2 gata4* (**F).** Blue arrowheads: earliest detected expression in either the cortex or epidermis; White arrowheads: approximate end of the MZ. Scale bars, 20 µm. *p < 0.05, **p < 0.01 two-sided Student’s t-test. For replicate numbers and specific *p*-values, please see the Source Data file.

To distinguish the directionality of GATA TF action on predicted targets, we next performed RNA-seq analyses on Arabidopsis root culture protoplasts transiently expressing GATA2 or GATA4 (Supplementary Data 4). Of the DEGs identified (q < 0.05, Log2 FC > 0.6), ~78% are affected upon both GATA2 and GATA4 expression, highlighting the high functional redundancy between these two TFs. Moreover, supporting the strength of the target prediction, expression of ~47% of predicted targets responds to one or both GATA TFs, greatly exceeding random expectations (hypergeometric test, 3,2E-43). Expression of the vast majority of the DEGs is upregulated, but ~22% are downregulated, indicating a duality of action as is common among TFs^3,45–48^. The combined expression profile of downregulated targets is anti-correlated with the expression dynamics of their putative GATA regulators, showing maximum expression in cells of the early MZ and in fully differentiated cells (Supplementary Fig. 4B). Contrary to this, the combined expression profiles of upregulated SF1 GATA targets closely follows that of *GATA2*, *GATA4*, as well as *GATA12*, in both the scRNA-seq atlas and along the cortex terminal trajectory (Fig. 4A). These targets show maximum expression in cells positioned between the proliferating cells at the core of the atlas UMAP and the more-mature tissues at the UMAP edges, as well as in the late and post MZs of the cortex trajectory (Fig. 4A). Likewise, similar profiles are seen for accessibility of both the SF1 motif and chromatin at predicted targets in the scATAC-seq atlas and cortex trajectory (Fig. 4B, Supplementary Fig. 4C). These observations are also seen when examining expression/accessibility profiles within independent root scRNA-seq and scATAC-seq atlases^14,25^ (Supplementary Fig. 4D, E), further supporting the target predictions.

Among the targets are genes such as *PRL*, *SCZ*, *ARF16*, *CDC6*, and several other cyclins that regulate cell proliferation in the early MZ (Supplementary Data 1, 4), consistent with a role for the SF1 GATA TFs in promoting the exit from cell proliferation. Moreover, given that nearly 80 percent of targets responding to GATA2 or GATA4 expression are upregulated, the SF1 GATA TFs appear to play a more prominent role in activating genes linked to the onset of differentiation. This is in line with the in silico prediction that perturbation of SF1 GATA activity delays progression toward differentiation (Fig. 3C). Several lines of evidence further support this idea. Nearly 57% of DEGs defining the late MZ in the trajectory are predicted SF1 GATA targets, and ~26% of the post MZ DEGs are predicted targets, well exceeding random expectations (p = 1.13E-19 and 7.8E-14, respectively, Supplementary Fig. 4F, Supplementary Data 1, 4). Strongly enriched GO terms for predicted targets include those associated with cell wall- and cytoskeleton-reorganization, processes associated with cell growth and differentiation (Supplementary Data 4). Further, among the SF1 GATA targets are numerous BR- and CK-signaling genes, including *BES1, BEH2, ARR1*, and *ARR12*, as well as downstream response genes known to promote cell elongation and differentiation^16^ (Supplementary Data 4). In addition, transient dual luciferase reporter assays, which can discern the effects of individual TF-promoter interactions within complex gene regulatory networks, validate direct positive regulatory activity of SF1 GATA TFs on the promoters of nine predicted targets selected for expression in the late or post MZ. Promoters of *BEH2* and *BAM8*, TFs that interact with the BR-signaling pathway, as well as seven additional predicted targets were significantly induced upon GATA4 expression (Supplementary Fig. 4G, Supplementary Data 5). Five of five target promoters tested showed a comparable response to GATA2 expression (Supplementary Fig. 4G). Finally, promoter-reporter fusions for ten additional predicted targets confirmed expression commencing from the late MZ or post MZ (Fig. 4C, Supplementary Data 5).

Consistent with the machine-learning prediction, these findings place the SF1 GATA TFs in an upstream, positive regulatory position to a large number of genes associated with cell elongation and differentiation. To a lesser extent, they also play a repressive role on genes associated with functions in the QC and early MZ. Therefore, as a definitive test of the in silico prediction that SF1 GATA TFs promote the transition from cell proliferation to differentiation, we perturbed SF1 GATA activity *in planta*. Prolonged ectopic activation of GATA4 largely halts primary root growth. Compared to mock treated *p35S:GATA4-GR* roots, primary root length and meristem length increase minimally over 48 h of induction, while the number of cells within the MZ declines from an average of ~34 in mock treated roots to ~10 in GATA4-induced roots (Fig. 4D, E). Moreover, CRISPR-mediated knockout of *GATA2* in a *gata4* mutant background (SALK_014511) results in a significant increase in primary root length, root meristem length, and MZ cell number (Fig. 4D, F, Supplementary Fig. 6A–C). This phenotype is comparable to that observed for *arr12*^40^, and confirms a delayed transition from cell proliferation to differentiation. Within this genotype, expression of the predicted targets *RGI3*, *AT1G61170*, *CML39*, *AT5G05220* and *MAPKKK15* is hugely reduced and/or displaced shootward away from the root tip (Fig. 4C). Changes in reporter expression are seen across cell files, suggesting that the SF1 GATA TFs coordinate the proliferation-to-differentiation transition in multiple tissue layers (Fig. 4C). The strength of these phenotypes is of note, given the potential for redundancy with GATA12 (Supplementary Fig. 3G), as well as potentially other more-broadly expressed GATA TFs, and underscores not only the predictive power of the machine learning framework, but the necessity of SF1 GATA TFs in regulating the timely transition from cell division to cell differentiation in the root.

### GATA TFs promote developmental progressions in a network integrating auxin and cytokinin signaling

The observed role in coordinating the switch from proliferation to differentiation presents the enticing possibility that the SF1 GATA factors sit within a broader gene regulatory network proposed to govern the mutually antagonistic actions of auxin and cytokinin in shaping the developmental structure of the root^15,16^. An auxin maximum at the SCN positions a PLT protein gradient that promotes cell division at the high-end of the gradient^30,31^. A drop in PLT levels higher up the meristem prevents cell division and permits expression of CK response regulators such as *ARR12* and *ARR1*^40^. The activation of these ARR-B TFs promotes cell elongation and differentiation and inhibits cell division, at least in part by downregulating PLT expression^40^.

**Fig. 5:**
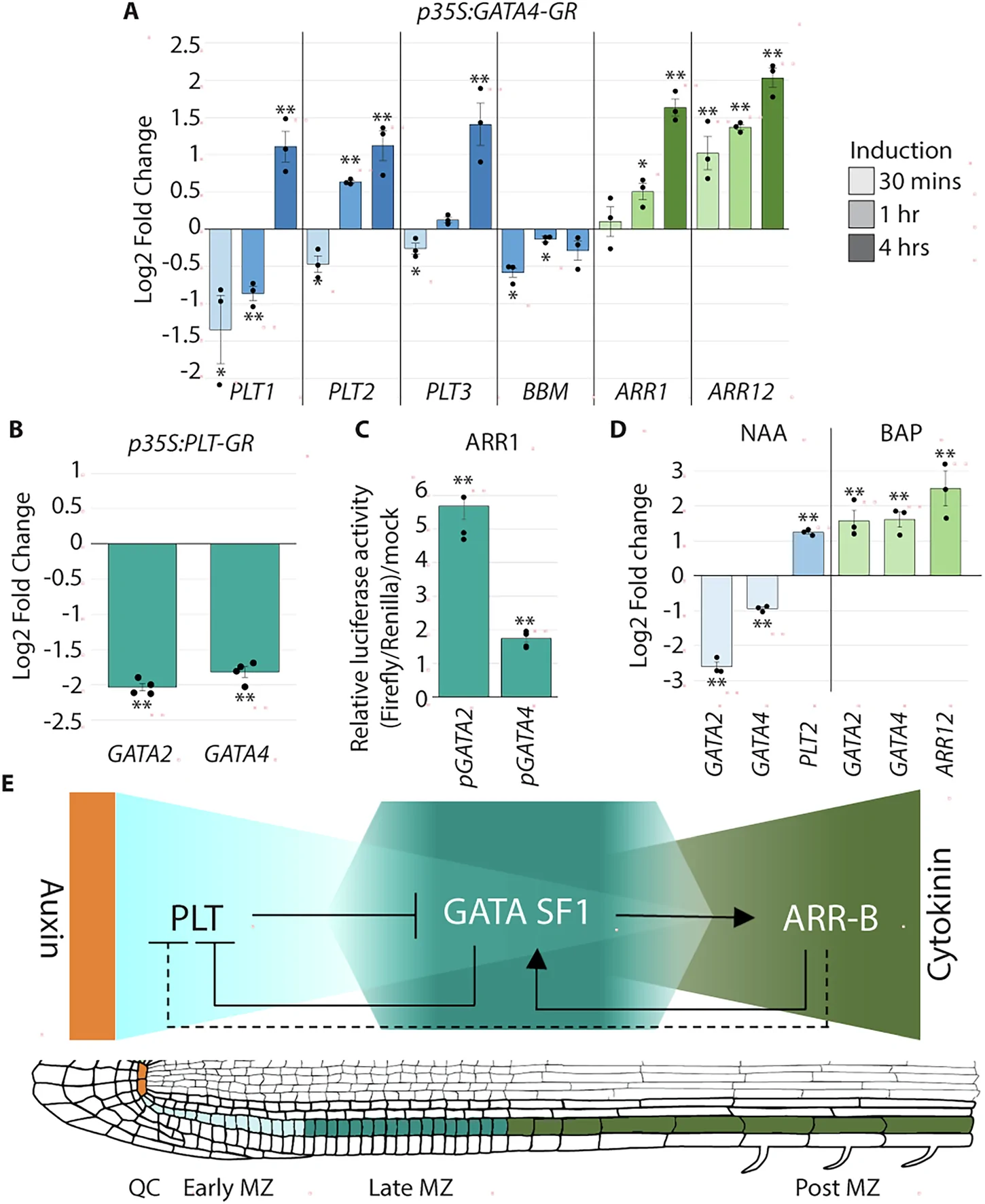
Model for root meristem size regulation by SF1 GATA TFs. **A** RT-qPCR analysis shows relative transcript levels (mean ± sd, *n* = 3 biological replicates) for several PLT genes decrease upon short-term GATA4 induction (*p35S:GATA4-GR*, 30 min 20 µM dexamethasone) but increase upon prolonged (4 hrs) induction. In contrast, transcript levels for *ARR1* and *ARR12* increase over time upon the same GATA4 induction. **B** RT-qPCR analysis shows relative *GATA2* and *GATA4* transcript levels (mean ± sd, *n* = 4 biological replicates) decrease significantly upon PLT2 induction (*p35S:PLT2-GR*, 2 hrs 20 µM dexamethasone). **C** Relative luciferase activity (mean ± se, *n* = 5 biological replicates), calculated as Firefly over Renilla luciferase activity, shows significant induction of *pGATA2:LUC* and *pGATA4:LUC* by ARR1. Values shown are relative to mock (no TF). **D** RT-qPCR analysis shows relative *GATA2* and *GATA4* transcript levels (mean ± sd, *n* = 3 biological replicates) decrease significantly in roots treated with exogenous auxin (24 hrs, 10 µM NAA), and increase upon application of CK (24 hrs, 10 µM BAP). As controls, *PLT2* and *ARR12* expression is significantly increased following NAA and BAP application, respectively. **E** Model for SF1 GATA TF-driven meristem size governance in the Arabidopsis root. SF1 GATA TFs fit into a GRN that coordinates the antagonistic effects of auxin and CK on cell proliferation and differentiation through interacting reinforcing feedback loops. Expression of the GATA genes is repressed by the auxin-responsive PLT TFs and induced by the CK-response regulators, ARR1 and ARR12. The SF1 GATA TFs, in turn, promote expression of *ARR1* and *ARR12*, and repress *PLT* expression, including potentially indirectly via the ARR-B TFs. Solid lines: validated direct interactions; Dashed lines: predicted and/or indirect interactions. *p < 0.05, **p < 0.01 two-sided Student’s t-test. For replicate numbers and specific *p*-values, please see the Source Data file.

Notably, both *ARR1* and *ARR12* are among the predicted SF1 GATA targets. In addition, *PLT2* and *BBM* are predicted targets, and binding sites for both GATA4 and GATA12 are distributed across the *PLT1, PLT3*, and *PLT7* genes, albeit that these are positioned outside detected open chromatin in the 1 kb promoter region (Supplementary Data 4). The ARR-B genes are activated by transient SF1 GATA expression in root protoplasts (Supplementary Data 4). In addition, *ARR1* and *ARR12* transcript levels increase significantly upon short-term induction of GATA4 in *p35S:GATA4-GR* seedling roots (Fig. 5A). Both observations identify GATA2 and GATA4 as activators of these ARR-B genes, consistent with their expression commencing in the late MZ^40,41^ (Supplementary Fig. 3G). Contrary to this, the expression patterns of *PLT1*, *PLT2, PLT3*, and *BBM* in the early MZ suggest the SF1 GATA TFs function as repressors of these PLT genes during root development^30^ (Fig. 3C–E). Indeed, *PLT3* and *PLT7* are downregulated upon transient *GATA2* or *GATA4* expression (Supplementary Data 4). Moreover, short-term induction of GATA4-GR resulted in the rapid downregulation of *PLT1, PLT2, PLT3*, and *BBM* transcript levels (30 min, Fig. 5A). Interestingly, consistent with previously proposed incoherent feedback regulation from the post MZ on genes expressed in the SCN^49,50^, the repressive response of PLT was only transient and transcript levels for the PLT genes increased upon prolonged ectopic GATA4 activation (Fig. 5A).

To further examine how the SF1 GATA TFs fit into the regulatory network balancing the effects of auxin and cytokinin on cell division and differentiation, we also explored links from PLT and ARR-B TFs onto expression of SF1 GATA genes. Both *GATA2* and *GATA4* are among PLT targets predicted by ChIP-seq^32^, and RT-qPCR shows that transcript levels for both genes are downregulated upon short-term ectopic activation of PLT2 (2 hrs, *p35S:PLT2-GR*, Fig. 5B, Supplementary Fig. 6D). This effect mirrors the repressive action of PLT2 on *ALTERED PHLOEM* (*APL*), a MYB TF that guides differentiation in the phloem^17^, and contrasts to the activation of targets such as *HANABA TARANU* (*HAN)*, *YUC3*, and *TLP8*, the first of which co-expresses with PLT in the early MZ of the cortex^30,32^ (Fig. 3C, Supplementary Fig. 6E). It follows that PLT TFs act as repressors on the SF1 GATA genes, thereby delaying expression until PLT levels decline away from the SCN in the late MZ. *GATA2* and *GATA4* are also predicted targets of the ARR-B TFs^40,51^. Although we were unsuccessful in generating inducible lines for ARR1 and ARR12, dual luciferase assays show direct activation of both the *GATA2* and *GATA4* promoters by ARR1 (Fig. 5C). An equivalent analysis for ARR12 action was unfortunately not possible as a functional copy of ARR12 again proved impossible to clone. Further supporting the respective actions of PLT and ARR-B TFs on the GATA genes, RT-qPCR of seedling roots found *GATA2* and *GATA4* to be strongly repressed upon application of exogenous auxin and induced upon CK treatment (Fig. 5D). These observations together imply that expression of *GATA2* and *GATA4* in the late MZ reflects the opposing activities of auxin-responsive PLT TFs on the one side and CK-responsive ARR-B TFs on the other. Taken together, the data reveal that SF1 GATA TFs are positioned within a GRN between PLT and ARR-B TFs to coordinate the timed transition from cell proliferation to cell elongation and differentiation.

## Discussion

For an organ composed of multiple tissues to develop properly, lineage-specific transcriptional dynamics that drive cell type specialization must coordinate with general maturation processes. This imposes the need for precise spatiotemporal regulation of gene expression at both the tissue and organ scales. In metazoan systems, paused RNA polymerases prime genes for rapid, synchronous activation in response to signaling cues to ensure the precise timing of gene expression during cell state transitions^52,53^. However, as a direct equivalent of RNA polymerase pausing is tenuous in plants, the question emerges of what alternative mechanisms may compensate to coordinate dynamic developmental programs. Here, by examining single-cell resolution developmental trajectories depicting transcriptomic- and chromatin dynamics, we reveal phased transcriptomic landscapes (perhaps akin to Waddington’s epigenetic landscape1) underlying the path from stem cell through early differentiation, within which chromatin accessibility marks the expression of key developmental regulators. Among them are the SF1 GATA TFs, which integrate inputs from multiple cues to ensure temporal precision in the progression of root development.

Previously, the correlation between gene expression and chromatin accessibility has been described as generally weak^23,54,55^. While this holds true at a global scale, scrutiny of RNA-seq and ATAC-seq data at the single-cell level provides a more nuanced perspective. At the atlas level, UMAP representations of scRNA-seq and scATAC-seq data closely resemble each other, both reflecting the spatiotemporal organization of cell types and maturation states in the root. This suggests that chromatin accessibility is, in fact, a strong predictor of transcriptional programs differentiating root cell types and states. Specifically, considering GT trajectories, expression of key upstream regulators of cell proliferation, such as the PLT and HDG TFs, is governed by chromatin accessibility. Likewise, many genes associated with cell identity and differentiation processes are regulated by modulating chromatin accessibility. This contrasts with the gene expression profiles of cells in the early MZ, which are mechanistically associated with cell proliferation. Chromatin states at these genes appear largely preset and stable along the trajectory. As a result, the cortex and endodermal lineages become distinct on a chromatin level in near parallel with their physical separation. However, transcriptomic differentiation between these lineages is clarified only in the late MZ, when the shared cell division-linked gene expression program ends. Thus, although the regulation of gene expression is undoubtedly more complex than a simple binary change in chromatin accessibility, expression of a disproportionately large number of key developmental regulators correlates well with chromatin state, suggesting that chromatin accessibility may serve to prioritize functionally important factors. To this end, it will be well worth examining the chromatin level changes distinguishing the cell lineages immediately at the SCN and beyond.

The phased gene expression profile of the GT trajectory indicates an abrupt switch between the transcriptional programs of the transit amplifying cells in the early MZ and the cells in the post MZ commencing cellular differentiation. This switch in gene expression identifies a regulatory ‘hub’ within late MZ cells. Within these cells and in cells of the post MZ, considerable chromatin rearrangement underpins the activation of differentiation programs, and to a notable though lesser extent, active repression of cell proliferation. DARs distinguishing cells of the late MZ show an enrichment of GATA SF1 TF binding motifs, in line with expression of *GATA2* and *GATA4* in these cells and into the elongation zone. Whether these GATAs act as pioneer TFs, as reported for GATA factors in mammalian systems^56,57^, and thereby actively contribute to the chromatin reorganization observed in the late MZ, remains an open question. However, the broad functional spectrum of the SF1 GATA TF targets positions them as key regulators coordinating developmental progression in the GT as well as beyond. They integrate intricate hormonal cues to time general maturation in the root (Fig. 5E), the functional significance of which is underscored by the strong alterations in root meristem size upon perturbation of these GATA TFs.

The regulatory action of the SF1 GATA TFs fits well into an established model for root meristem size control, in which PLT and ARR-B TFs mediate the mutually antagonistic effects of auxin and CK on cell proliferation and differentiation^16,40,58^. We propose an extended model wherein PLT TFs act as direct repressors of SF1 GATA genes, limiting their expression to the late MZ of the root (Fig. 5E). Conversely, the SF1 GATA TFs repress *PLT* expression, thereby promoting the exit from cell division in the root. In addition to this direct regulatory interaction, negative feedback regulation from the SF1 GATA TFs onto *PLT* expression may be reinforced indirectly via the transcriptional activation of *ARR12* and *ARR1*, which have been implicated in repressing PLT genes^40^. Although technical limitations preclude analysis of ARR12 regulation of SF1 *GATA* promoters, the observed activation of SF1 GATA expression by ARR1 and by CK treatment points to a second feedback loop in the regulatory network. Notably, the SF1 GATA TFs promote expression of *ARR1* and *ARR12*, which in turn promote expression of the GATA genes. As such, the SF1 GATA TFs connect a reciprocal repressive regulatory loop to reciprocal positive feedback regulation, creating a reinforced bistable switch that would ensure a sharp, robust transition from cell proliferation to differentiation^59,60^.

Together, our study establishes a new mechanistic framework for how auxin and CK signaling are integrated to regulate root meristem size. It identifies the SF1 GATA TFs as principal transcriptional regulators at the tipping point of a bistable system, ensuring developmental precision and robustness in timing the cell proliferation-to-differentiation switch. From it, several intriguing questions emerge. First and foremost, do the SF1 GATAs act as pioneer TFs^56,57^, triggering the observed chromatin changes rather than responding to them? Second, given that the SF1 GATA TFs show slight variations in the onset and strength of expression across tissue layers (Fig. 3D, E), does the system allow for tissue-specific adjustments of meristem size? Third, because a subset of targets is expressed in a tissue-specific manner (Supplementary Data 1, 4), do SF1 GATA TFs coordinate lineage-specific differentiation programs within the general maturation system described above? Finally, giving the remarkable diversity of root expression profiles across the broader GATA TF family. Supplementary Fig. 3G, Supplementary Data 1^20,41,61,62^), what is the interplay between distinct GATA family members and what additional developmental processes do they control?

Beyond the specific findings, this work illustrates the depth of mechanistic insight attainable from multi-layered single-cell data when examined in a critical, biologically informed manner, and particularly demonstrates the power of machine learning-based network inference therein. To maximize the utility of the data generated in this study, we developed a tailored scATAC-seq online browser to complement our scRNA-seq data browser^63^ (https://www.zmbp-resources.uni-tuebingen.de/timmermans/plant-single-cell-browser/). Users can interrogate the data, including the developmental trajectories, through perspectives of ‘gene activity,’ ‘motif activity,’ and ‘peak activity,’ each reflecting distinct aspects of chromatin accessibility in a highly informative and intuitive format. Additionally, processed datasets are available for download to support further independent analyses, and a concise user manual is provided to facilitate effective use of the platform.

## Methods

### Plant growth

All analyses were performed in the *Arabidopsis thaliana* Columbia (Col-0) ecotype. Plants were grown at 22 °C on 1 % agar plates containing 0.5 x Murashige and Skoog (MS) medium (Duchefa Biochem, unless otherwise stated. Plants were grown under long-day conditions (16 hours light, 8 hours dark) following stratification for three days at 4 °C.

### Nuclei isolation

Seedlings were grown vertically on 1.4 % agar plates. Roots of six day old seedlings were cut 0.5 - 0.7 cm from their tip and a total of 180 mg of root tissue was collected into 2.5 ml high-density nuclei isolation buffer (HD-NIB; 1.7 M Sucrose, 10 mM MES/KOH pH 5.5, 10 mM NaCl, 10 mM KCl, 0.1% v/v 2-mercaptoethanol, 0.1 mM Spermine, 0.5 mM Spermidine, 1% w/v BSA, 0.2 % v/v Tween-20, 1 x EDTA-free cOmplete (Sigma), adapted from^64^). Tissue was rapidly chopped with a razor blade for 10 minutes and consecutively filtered through 100 µm and 40 µm nylon sieves. The homogenate was diluted with 2 ml fresh HD-NIB, and following centrifugation (40 min, 20,000 g), the pellet was resuspended in 150 µl NIB (as above but with 0.2 M Sucrose) supplemented with 0.5 % v/v Triton X-100 to dissolve plastid and mitochondrial organelles. The solution was subsequently diluted with NIB up to 2 ml. Following a second centrifugation (15 min, 350 g), the nuclei-enriched pellet was resuspended in 80 µl of 1x Diluted Nuclei Buffer, as provided in the 10X Genomics Chromium Single Cell ATAC-seq (scATAC-seq) kit. 10 µl of the final resuspensions were stained with DAPI for microscopic inspection and for counting of nuclei by hemocytometer.

### Single cell ATAC-seq library preparation and sequencing

Single-cell ATAC-seq libraries were produced from freshly isolated nuclei in accordance with the 10X Genomics Chromium Single Cell ATAC-seq kit protocol (v 1.1 protocol, Rev D), targeting ~14-15,000 nuclei per reaction. The adapter-ligated libraries were amplified using nine PCR cycles. Final library size and quality was checked on a DNA High Sensitivity Bioanalyzer chip (Agilent), and libraries were quantified using the NEBNext Library Quantification Kit for Illumina. Sequencing was performed on the NovaSeq (Illumina) platform using parameters recommended in the 10X Genomics protocol (150 bp Paired End reads, ~300 M sequenced read pairs per sample).

### Construction of Plasmids and Transgenic Lines

Guide RNAs (gRNAs) for the CRIPSR-mediated mutagenesis of *GATA2* were designed using the CRISPR/Cas9 tool from Benchling (https://benchling.com). Three gene specific gRNAs were designed within the first or second exon of *GATA2* (GAAGTCATAGTTCCTCCTAA, CCAAAGAAAGAACAATCCGG and CCAAAGAAAGAACAATCCGG). Modules containing the gRNAs were introduced by GreenGate cloning into vector *pEC:CAS9* (VIB Ghent), containing a FAST-RED (*pOLE1:OLE1_tagRFP*) cassette. Final constructs were transformed into a homozygous *gata4* T-DNA insertion line (SALK_014511). Mutations in *GATA2* were identified by sequence analysis of T1 seedlings. Three T2 families from a line carrying a 10 bp deletion allele of *GATA2* were used for phenotypic analysis and showed identical results.

A GATA4 inducible overexpression cassette (*p35S:GATA4-GR*) was assembled using GreenGate cloning. The *GATA4* gene body was amplified from cDNA (see Supplementary Data 5 for primer details), cloned in frame upstream of the DEX-inducible glucocorticoid receptor (GR), and inserted downstream of the CaMV35S promoter within a pCAMBIA vector containing a FAST-RED selection cassette^4^. The final construct was transformed into the Col-0 background. *GATA4* overexpression was assessed in seedling roots of independent T2 lines using the DEX-induction and RT qPCR protocols described above.

Fluorescent reporter cassettes were assembled using GreenGate cloning. For promoter reporter constructs, promoter fragments of ~3 kb were PCR amplified (see Supplementary Data 5 for primer details) and introduced as an entry module upstream of 2xYPET or 2xmCherry, a nuclear localisation signal (NLS), an OCS terminator, and a FAST-RED or FAST-Green selection cassette, into pCAMBIA modified to be compatible with the GreenGate system^4^. For protein fusion constructs, entry modules with the gene body and terminator of the gene of interest amplified by PCR, replaced the NLS and OCS terminator modules, respectively. All reporter constructs were transformed into the Col-0 background. Select reporter constructs were also introduced into the *gata2 gata4* or *p35S:GATA4-GR* backgrounds. For dual-reporter analyses, *pGATA2:2xmCherry-NLS* was introduced into selected verified reporter lines.

### Dexamethasone (DEX) Induction

Three- or five-day-old seedlings (for 48 h or 2-hour inductions, respectively) were transferred to fresh 0.5x MS plates containing 10nM or 20nM DEX alongside mock controls. Roots were harvested for RNA extraction or quantitative analyses of root traits after five-days total growth. Cell length in the cortex lineage was used to indicate the end of the meristem zone.

### Exogenous Auxin and Cytokinin Treatment

Col-0 seedlings were grown for five days before being transferred to 0.5x MS plates containing 10 µM 1-Naphthaleneacetic Acid (NAA, Sigma) or 6-Benzylaminopurine (BAP, Roch). After 24 h, 1cm root tips were dissected, and RT-qPCR was performed.

### Reverse transcription quantitative PCR

Roots were harvested, snap-frozen in liquid nitrogen, ground to a powder, and RNA extracted with Trizol reagent (Thermo Fisher Scientific) following the recommended protocol. RNA samples were DNase-treated using the TURBO DNA-free kit (Thermo Fisher Scientific) as per the manufacturer’s protocol. RNA samples were quantified by Nanodrop and quality assured based on RNA Bioanalyzer chip traces (Agilent). cDNA was generated from 0.5 – 1 µg RNA using the LunaScript RT SuperMix Kit and Oligo dT primers (NEB) following the recommended protocol. Quantitative PCR (qPCR) was performed using the Luna Universal qPCR Mix (NEB). *ELONGATION FACTOR1* (*EF1*) was used as normalization control (see Supplementary Data 5 for primer details).

### Confocal Imaging

Roots of five to seven-day-old seedlings were mounted on slides in 10 mg/mL Propidium Iodide (PI, Sigma-Aldrich) and imaged using either a Leica TCS SP8 with Resonant Scanner or a Zeiss LSM880 Airyscan confocal laser-scanning microscope. Images at the Zeiss microscope were acquired as follows: for YPET, excitation at 514 nm and image acquisition at 517-571 nm; for PI, excitation at 561 nm and image acquisition at 589-718 nm. Images at the Leica TCS SP8 were acquired with the following settings: for YPET excitation at 514 nm and image acquisition at 520-555 nm using Hybrid detectors; for PI, excitation at 561 nm and image acquisition at 570-630 nm with a standard photomultiplier tube. Images were processed with ImageJ/Fiji (http://rsb.info.nih.gov/ij/) as well as Adobe Photoshop v25.12.2 (Adobe Systems), for qualitative image presentation. Color merges were performed with ImageJ, and figures were assembled with Adobe Illustrator v29.4. For each analysis, multiple (n⩾3) independent events per construct/background were analysed.

### Transient luciferase assay in arabidopsis root culture protoplasts

To generate Luciferase reporter plasmids, ~3 kb promoter fragments (see Supplementary Data 5 for primer details), defined relative to the start codon, were amplified from Col-0 genomic DNA, and cloned into pLucTraP^65^ to drive expression of Firefly Luciferase. Effector plasmids expressing GATA4 or GATA2 were generated by cloning coding sequences, amplified from Col-0 cDNA, behind the *2x35S* promoter in pJIT6041. Root culture protoplasts were isolated and transfected as previously described^4^. Protoplasts were co-transfected with 1 µg each of reporter (pLucTraP) and effector (pJIT60) plasmid along with 1 ug pGL4.70 plasmid expressing Renilla Luciferase from the *2x35S* promoter as an internal control. Transfected protoplasts were incubated overnight at room temperature in the dark and then processed and analysed using the Dual-Luciferase Reporter Assay System (Promega) as previously described^65^. Luminescence was measured using the Tecan’s Infinite F200 plate reader. Relative Luciferase activity was calculated as Firefly over Renilla luciferase activity. Statistical significance was calculated using a two-sided Student’s t-test.

### Transient GATA overexpression analysis

7 µg GATA effector plasmids used for the dual-luciferase assay (above) were co-transfected into root culture protoplasts with *p35S:2xNLS-GFP*, as described above. Transfections with *p35S:2xNLS-GFP* alone were used as a negative control. Following filtration (70 µm), protoplasts expressing GFP were sorted into Trizol LS reagent (Thermo Scientific) with a Modular Flow Cytometer (2007, Backman-Coulter). Scatter, auto-fluorescence, and GFP were excited using a solid-state laser at 75 mW. Singlet cells were identified based on proportional FCS-LogArea to SSC-LogArea scatter and clumped cells excluded by gating on SSC-LogArea vs SSC-width. GFP positive cells were identified by comparing peak (FL1; 534/30) GFP emission to shoulder emission (FL3; 620/29). Sorts were performed with a 70 µm nozzle at 61.0/30.0 psi (sample/sheath) pressure differential; sheath 1× PBS at pH 7.0. Samples were processed in four replicates, and 60,000 cells were collected for each. RNA was extracted as described above. Isolated RNA was quantified using a Quantus Fluorometer (Promega), and quality assessed using a RNA Bioanalyzer (Agilent). RNA libraries were prepared with the NEBNext UltraExpress® RNA Library Prep Kit (NEB) following standard protocols and sequenced to a depth of ~10 M reads per sample.

### Single cell RNA-seq analysis

The Arabidopsis root scRNA-seq data generated previously^12^ was reanalysed as follows: In total, 497 million reads from two replicates were processed with Cell Ranger (v7.1.0; 10X Genomics) using the TAIR10 reference genome and TAIR10.55 genome annotation (https://ftp.ensemblgenomes.ebi.ac.uk/pub/plants/release-55/gtf/arabidopsis_thaliana/). Downstream processing was done with the Seurat R package^66^ (v5.1.0). Cells with fewer than 500-, or more than 100,000 UMIs, were removed from subsequent analysis, as were genes detected in fewer than five cells. Further, cells containing more than 5 % reads mapped to mitochondrial genes were removed. Count matrices were normalized with the ‘SCTransform’ function, and replicates were integrated following Seurat’s integration pipeline using the canonical correlation analysis (CCA) method and 50 principal components. Genes previously identified as induced by the protoplasting procedure^12^ were excluded from the integration procedure. UMAP visualizations were generated using integrated dimensionality reduction, and cell clusters were identified with a resolution of 0.5. Clusters were annotated using tissue-specific marker genes as previously described^12^.

### Analysis of ground tissue development trajectories from transcriptomic data

Cell clusters C3 (meristematic), C9 (cortex), C12 (endodermis), and C13 (columella and QC) were isolated from the atlas based on the expression of tissue-specific marker genes. Cells were re-clustered (resolution = 1.5) and a new UMAP constructed (local.connectivity = 70, spread = 0.6). Subclusters derived from C3 that did not express columella/QC/GT markers and deviated from the observed columella-QC-GT continuum within the UMAP, were excluded from further analysis. To further refine the trajectory and remove potentially confounding cells from other tissue files, the QC cell-containing cluster was subclustered (resolution 0.5) and cells not expressing QC marker genes (*FLZ5*, *SCRM2*, *RGF1*, *TEL1*, *BBM*, *MYB56*, *AT3G13510*, *LSH3*, *FD*, *WOX5*, *SOT17*, Supplementary Data 6), or those expressing the epidermal factor, *WEREWOLF*, were excluded. A final UMAP (local.connectivity = 70, spread = 0.6) and cell clusters (resolution = 0.6) was calculated for the curated dataset. Clusters were manually annotated, and DEGs were calculated with the Wilcoxon Rank Sum test. Developmental trajectory and pseudotime analyses were performed on the curated dataset using the Monocle3 R package^67^ (v1.3.6) following standard pipelines except that for the ‘learn_graph’ function nn.k = 50 and ncenter = 150. DEGs along the trajectory were identified with the’graph_test’ function using the ‘kNN_graph’ transferred from Seurat. The linearized cortex- and endodermis-terminal trajectories were isolated with ‘choose_graph_segment’ by manually setting the root at the tip of the columella cluster and the termini at the cortex and endodermal tips of the branched trajectory, respectively. The Pearson correlation of transcriptomic similarity among trajectory cells (ordered by pseudotime values) was calculated using cell embedding values from the integrated dimensionality reduction. The ‘AddModuleScore’ function from the Seurat package was used for visualization of expression patterns of selected gene groups.

### Single cell ATAC-seq analysis

Read pairs were processed with Cell Ranger ATAC (v2.1.0, 10X Genomics) using the TAIR10 reference genome and TAIR10.55 genome annotation from Ensembl Plants. Peak files from the two replicates were combined with the ‘Reduce’ function from the GenomicRanges R package (v1.46.1) and the combined dataset subsequently processed using the Signac R package^68^ (v1.13.0). Genomic regions identified by^69^ as potentially providing an artificial signal were excluded from further analyses. To retain only the high-quality informative nuclei, those with read counts fewer than 4000 or more than 31,000, with less than 40 % reads in peaks, a nucleosome signal lower than 0.5, or a TSS enrichment lower than two, were removed from further analyses. The filtered data was normalized with the ‘RunTFIDF’ function and 50 % of features (peaks) with the highest read counts were selected. Singular value decomposition (SVD) was calculated on these features and the 2nd to 30th dimensions used for UMAP and cell clustering analyses with the LSI reduction method and the SLM clustering algorithm. Cluster specific peaks were identified with MACS2^70^ and called with the ‘Callpeaks’ function from Signac. Resulting peak sets were manually combined with the ‘reduce’ function (min.gapwidth = 0) to generate a comprehensive peak set with a resolution on a cluster level. This set was used for the construction of the new count matrix (assay), which was used for further analyses. DA peaks were identified with ‘FindAllMarkers’ using logistic regression. Cell identities were assigned to the clusters by manual inspection of gene activity scores calculated with the ‘GeneActivity’ function. The correlation between single-cell and bulk ATAC-seq data^24^ was calculated by summing up the fragments per genomic region and the Pearson-correlation coefficient calculated in R.

### Analysis of ground tissue development trajectories from chromatin accessibility data

GT developmental trajectories based on the scATAC-seq data were generated following a strategy analogous to that of the scRNA-seq GT trajectory. Clusters 2, 3, 6, 7, 12, 13, 16, and 17, containing nuclei of the columella, SCN, cortex, and endodermis, were extracted from the atlas, and a new UMAP was calculated as described above. Cluster C6, containing cells from the SCN, was subclustered with increased resolution (resolution = 0.5), and nuclei of epidermal or vascular origin, discerned based on their diverging position away from the cortex/endodermis terminal trajectory and their respective position within the overall UMAP, were removed. Terminal clusters of the cortex and endodermis branches without a transcriptomic counterpart were likewise removed. A final UMAP was calculated for this curated dataset with spread = 0.4 and n.neighbors = 50. Cell identities were estimated using label transfer from the transcriptomic data. Gene activity (Signac), considering 250bp upstream and downstream of the TSS, was calculated for the 3,000 highly variable features identified in the transcriptomic trajectory. The activity matrix was log normalized, scaled and used for the identification of the transfer anchors with ‘FindTransferAnchors’ (reduction = “cca”, dims = 1:10) and label transfer with ‘TransferData’ using LSI weight reduction (dims = 2:30). The trajectory was re-clustered with a high resolution (resolution = 2) followed with fine sub-clustering of selected clusters, and the cell identities were manually assigned based on the transferred scores. Differential accessible chromatin regions (DARs, Log2 fold change ⩾1 and an q < 0.05) were identified with the Wilcoxon Rank Sum test and genes proximal to DARs were defined as being located within a 2,500 bp radius from the peak center. Pseudotime values and the DARs across the trajectory were calculated with Monocle3, as described for the transcriptomic trajectory. For complimentary GLUE analysis, the Seurat objects of transcriptomic and chromatin trajectories were converted to anndata format with the Zellconverter package. Both objects were then preprocessed following the standard GLUE pipeline^27^. The guidance graph construction and model training were done with default settings. The cell identity labels were transferred from transcriptomic to chromatin trajectory with scglue.data.transfer_labels. The original UMAP previously generated with Seurat was used for visualization.

### GRN inference and in silico TF perturbation analyses

Cluster-specific GRNs were constructed using CellOracle^38^ (v0.20.0), with parameters set as suggested unless stated otherwise. The peak co-accessibility network was calculated with Cicero^71^ (v1.3.9) with s = 0.703, distance_constraint = 50,000, and window = 100,000 using the scATAC-seq GT trajectory, excluding the columella cluster. Only links with a co-accessibility score >0.8 were retained. Genes next to open chromatin regions were identified using TAIR10.55. TF binding motifs were obtained from CisBP3.0^72^. Active GRN edges were selected using the scRNA-seq GT trajectory, again excluding the columella cluster. The top 3,000 highly variable features were identified with Seurat’s FindVariableFeatures. KNN imputation was performed using 25 principal components. Within the resulting GRN, only the top 3,000 links per cluster were retained for downstream analysis. In silico gene perturbations were performed following the CellOracle manual. The results were visualized with min_mass = 6 and vm = 0.8. Expression values were set to 0 for loss-of-function simulations.

### Bulk RNA-seq analysis

Sequence read (paired-end, 150 bp) quality was checked with FastQC (v0.11.9), and adapter contamination was removed with fastp with options -q 30 -l 50 (v0.21.0)^73^. Reads were aligned to the reference transcriptome (TAIR10.51) with the STAR aligner^74^ (v2.7.9). Aligned reads were quantified with RSEM^75^ (v1.3.1). Count matrices were imported with the tximport R package and further analyzed with DEseq2^75,76^ following the standard pipeline (v1.32.0).

### GO enrichment analysis

Gene ontology (GO) enrichment analyses, based on biological processes, were performed with PANTHER version 19.0 (http://pantherdb.org) using standard parameters.

### TF binding site motif enrichment across the scATAC-seq GT trajectory

TF binding site motif enrichment in DARs for the individual GT trajectory clusters was calculated with HOMER^77^ (v5.1) with FindMotifsGenome.pl using a masked version of the TAR10 reference genome. To identify putative TF target genes, position frequency matrices obtained from the JASPAR 2024 database^29^ were mapped onto the accessible chromatin regions (ACRs) using FIMO from the MEME suite^78^ (v5.5.7), with a p < 1e-4 cut-off. The background model was calculated on all identified ACRs using ‘fasta-get-markov’. ACRs containing a motif of interest were retrieved, and gene promoters (1000 bp upstream of the TSS) containing the accessible motifs were identified. Motif accessibility was calculated using the ‘RunChromVAR’ function from the Signac package. The predicted target accessibility was summarized with the ‘AddModuleScore’ function applied to the gene activity values.

### Heatmap analysis

Heatmaps showing expression/accessibility across the course of pseudotime were constructed using the log-normalized and scaled expression matrices and TF-IDF-normalized accessibility matrices. The cells (columns) were reordered by pseudotime, binned into 100 bins, and the data were fitted with a cubic smoothing spline. Seven and five degrees of freedom were used for the transcriptomic and accessibility data, respectively. The features (rows) were clustered and manually reordered based on the maximal value position and plotted as z-scores using the ‘ComplexHeatmap’ package^79^.

## Supplementary information

Supplementary Information

Description of Additional Supplementary Files

Supplementary Data 1

Supplementary Data 2

Supplementary Data 3

Supplementary Data 4

Supplementary Data 5

Transparent Peer Review file

## Source data

Source Data

## Data Availability

Both scRNA-seq and scATAC-seq atlases presented in this work can be explored on our interactive browser^63^ (https://www.zmbp-resources.uni-tuebingen.de/timmermans/plant-single-cell-browser/. Bulk RNA-seq, scRNA-seq, and scATAC-seq data, both raw and processed files, can be downloaded from NCBI’s Gene Expression Omnibus under accession number GSE299784. Source data are provided with this paper.
