# Supplementary material for: GATA transcription factors coordinate developmental progression in the arabidopsis root
Source: Nat Commun. 2026 Nov 3;17:11367. doi: 10.1038/s41467-026-78787-0 (PMC13731660; doi:10.1038/s41467-026-78787-0)
Supplement: Supplementary file 1 [file 41467_2026_78787_MOESM1_ESM.pdf]

Supplementary Information

GATA Transcription Factors Coordinate Developmental Progression in the Arabidopsis Root

Tom Denyer, Pavel Solansky, Arvid Herrmann, Francesco de Martino, Efthymia Symeonidi, Valentin Brösamle, and Marja C. P. Timmermans

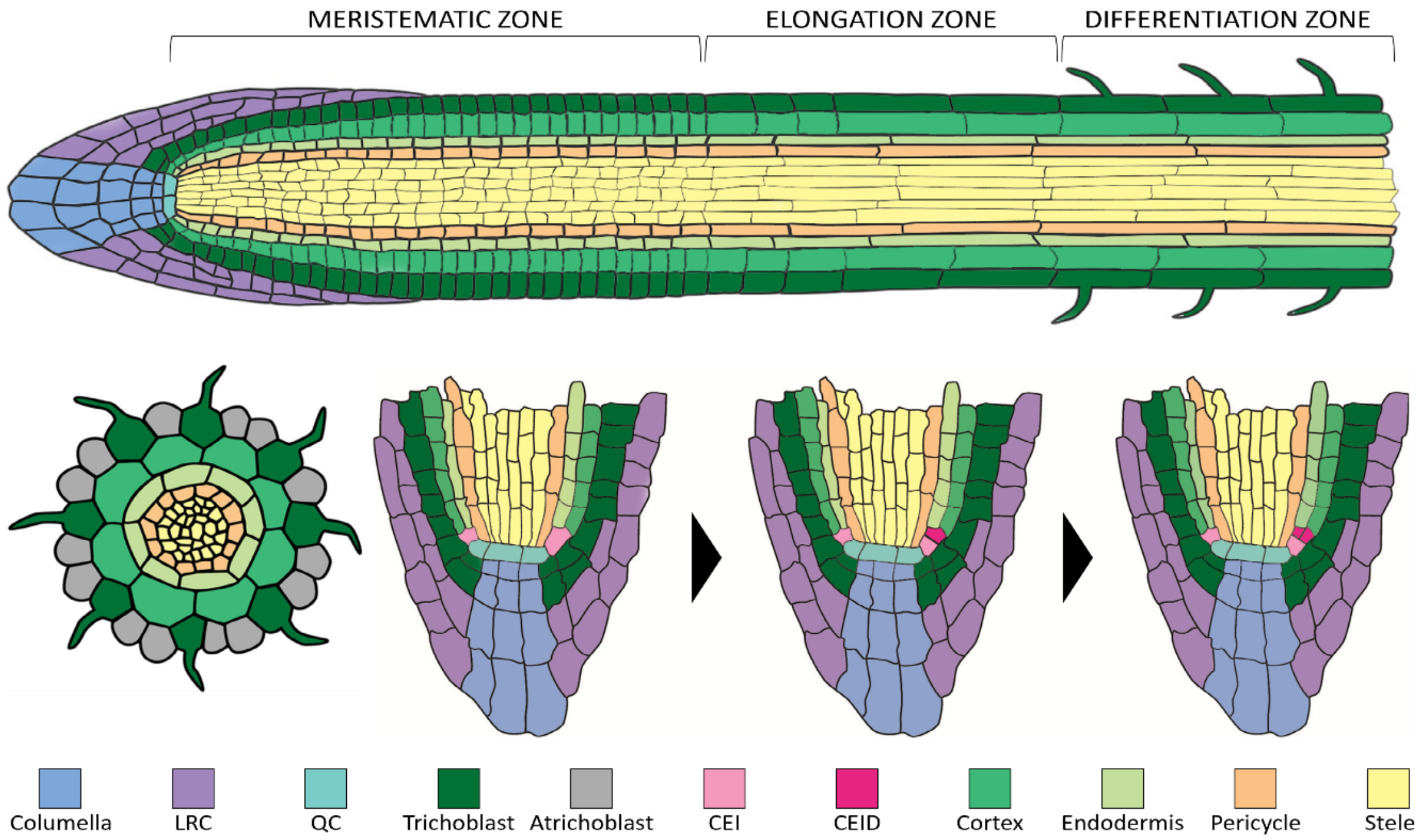

Supplementary Figure 1. Diagrams of the Arabidopsis root highlighting ground tissue development. (related to Figures 1 and 2). Developmental zones are captured along the longitudinal axis (top) while the root also shows radial symmetry with concentric rings of pericycle, endodermis, cortex and epidermis surrounding a central stele (bottom left). Bottom right: A focus on the stem cell niche (SCN) illustrating formation of the cortex and endodermis ground tissue (GT) cell files via successive asymmetric divisions of the GT initial (CEI). The CEI divides to produce a daughter cell (CEID), which upon division gives rise to cortex and endodermis cell files. Images adapted from Root illustrations – figshare collection (2018).

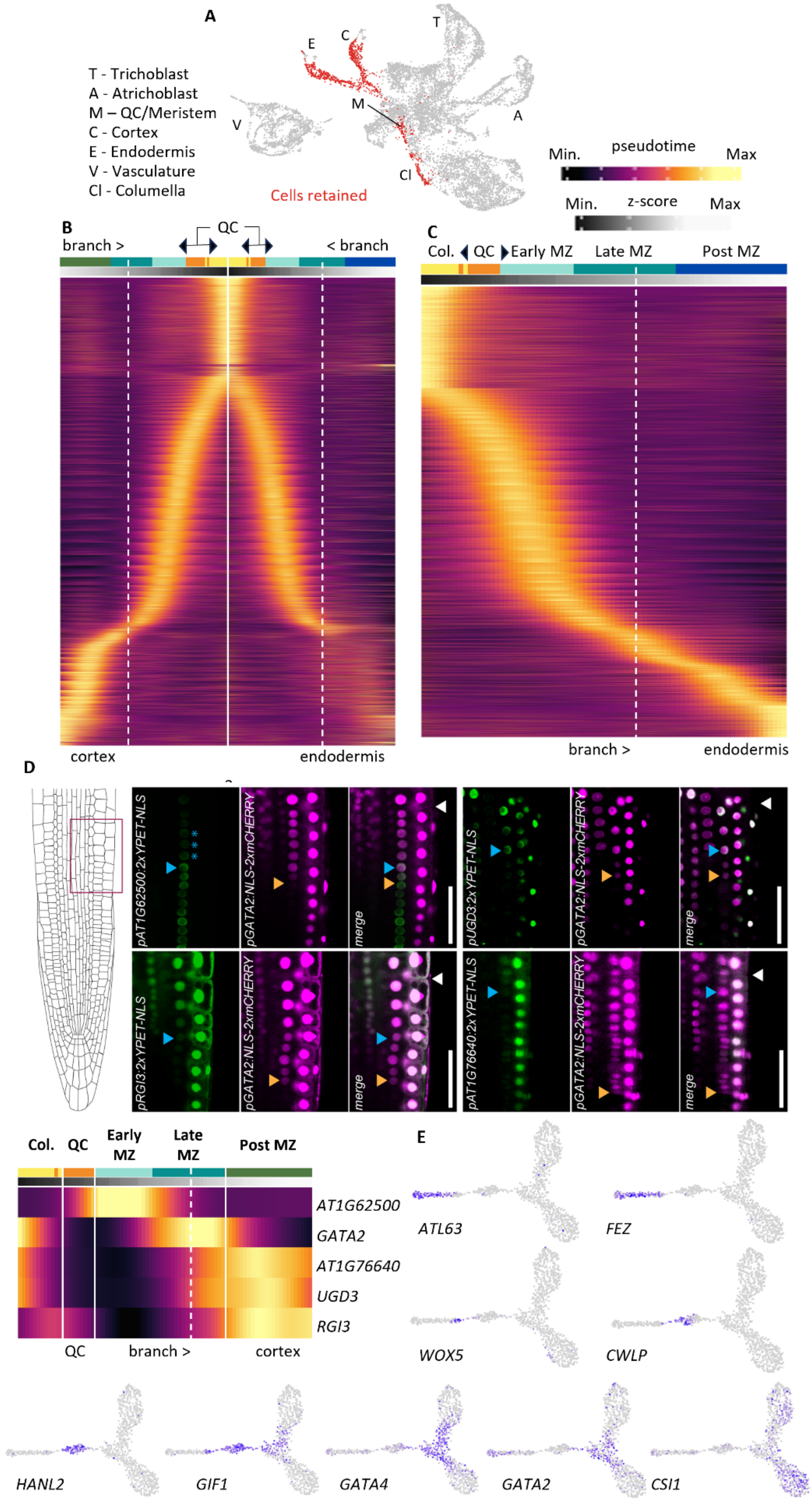

Supplementary Figure 2. A switch in gene expression profiles from cell division to differentiation marks the late MZ in the Arabidopsis root GT (related to Figure 1). (A) Cells selected for the GT developmental trajectory highlighted on the scRNA-seq atlas UMAP. **(B)** Heatmap of genes changing expression along the ground tissue trajectory reveals a switch in gene expression programs, occurring within cells of the late MZ region. Note that

the cortex and endodermal gene expression profiles are poorly distinguishable before this point. Cortex and endodermal trajectories progress left and right, respectively, from the QC regions. Arrows either side of the QC indicate bidirectionality of the trajectory from this point. **(C)** Heatmap (as in Fig. 1C) of genes changing expression along the endodermal-terminal trajectory. Dashed vertical lines: branch point in the GT trajectory; Top horizontal bars: distribution of cells along pseudotime coloured by cluster assignment. **(D)** Complementing Figs 1E-Q, confocal images of transcriptional reporters for select temporal marker genes in the root (green) co-expressed with *GATA2* (magenta), as a representative for expression commencing in the late MZ, illustrate the accurate resolution of the GT trajectories. Blue arrowheads: earliest/latest detected expression of the select reporter in the cortex; Orange arrowheads: earliest detected expression of *GATA2* in the cortex. Scale bars, 50 µm. Below left is a Heatmap (as in 1E) showing expression profiles of the marker genes along the cortex-terminal trajectory. **(E)** Expression of select marker genes (as Fig. 1E), shown on the branched GT trajectory UMAP (Fig. 1B).

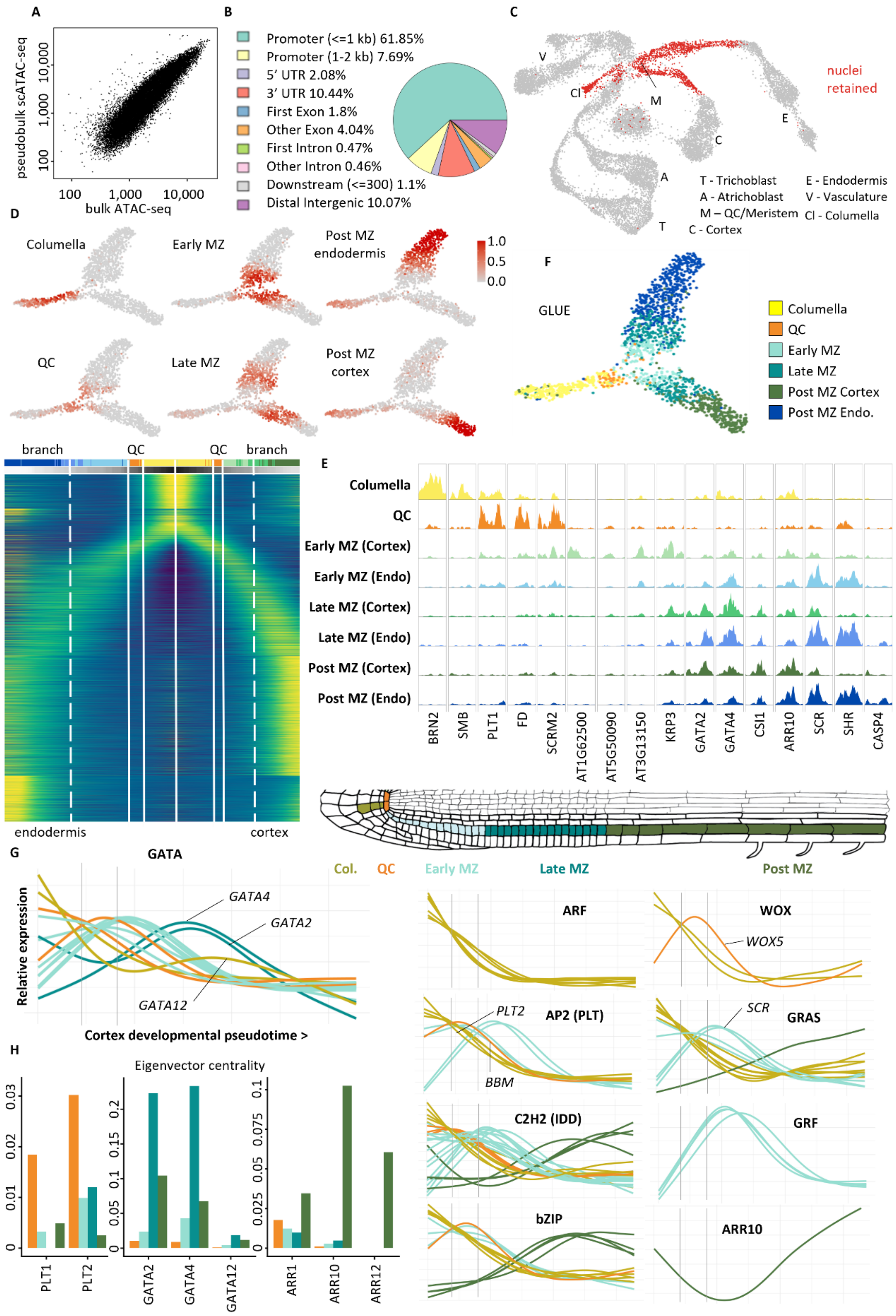

Supplementary Figure 3. Dynamic changes in chromatin accessibility underly Arabidopsis root GT development (related to Figure 2). (A) Comparative analysis of pseudo-bulked scATAC-seq data against bulk ATAC-seq data (Tannenbaum et al., 2018) shows a high Pearson's correlation, indicating the high quality of the scATAC-seq data. Axes show total fragment counts per genomic region. **(B)** Distribution of the positions of accessible chromatin regions relative to genes in the scATAC-seq root atlas. **(C)** Nuclei selected for the GT scATAC-

seq trajectory identified on the scATAC-seq atlas UMAP. **(D)** Top: Annotation of GT trajectory regions based on prediction scores from the equivalent scRNA-seq trajectory. Bottom: Chromatin accessibility heatmap for genomic regions that change accessibility along the GT trajectory show striking changes in chromatin accessibility associated with differentiation. Cortex and endodermal trajectories progress right and left, respectively, from the centre of the heatmap. Solid vertical lines: boundaries around the QC and MZ; Dashed vertical line: approximate point at which the scRNA-seq GT trajectory bifurcates; Top horizontal bar: distribution of nuclei along pseudotime coloured by cluster assignment. **(E)** Cluster-aggregated chromatin accessibility near known marker genes (columns) illustrating DARs defining distinct clusters of the GT trajectory (rows). Each column shows a region of 1,000 bp positioned within 2,5 kb of the gene body, in either direction, showing strong differential accessibility towards one-, or a subset of clusters. **(F)** GT trajectory region assignment based on GLUE. **(G)** Relative expression profiles of TFs from select (sub)families along the cortex-terminal trajectory. Individual profiles are coloured based on the zone in which a given TF shows maximum expression (colours as per the root image, right). Only TFs dynamically expressed along the trajectory are shown. x-axis: pseudotime progression of the cortex developmental trajectory; Vertical lines: position of the QC along pseudotime; y-axis: relative expression level. **(H)** Eigenvector centrality (EC) score indicating the relative importance of select genes within each cluster-specific GRN inferred from integrated scATAC-seq and scRNA-seq developmental trajectories. EC assigns higher scores to genes connected to other highly influential genes, thereby capturing network-wide influence.

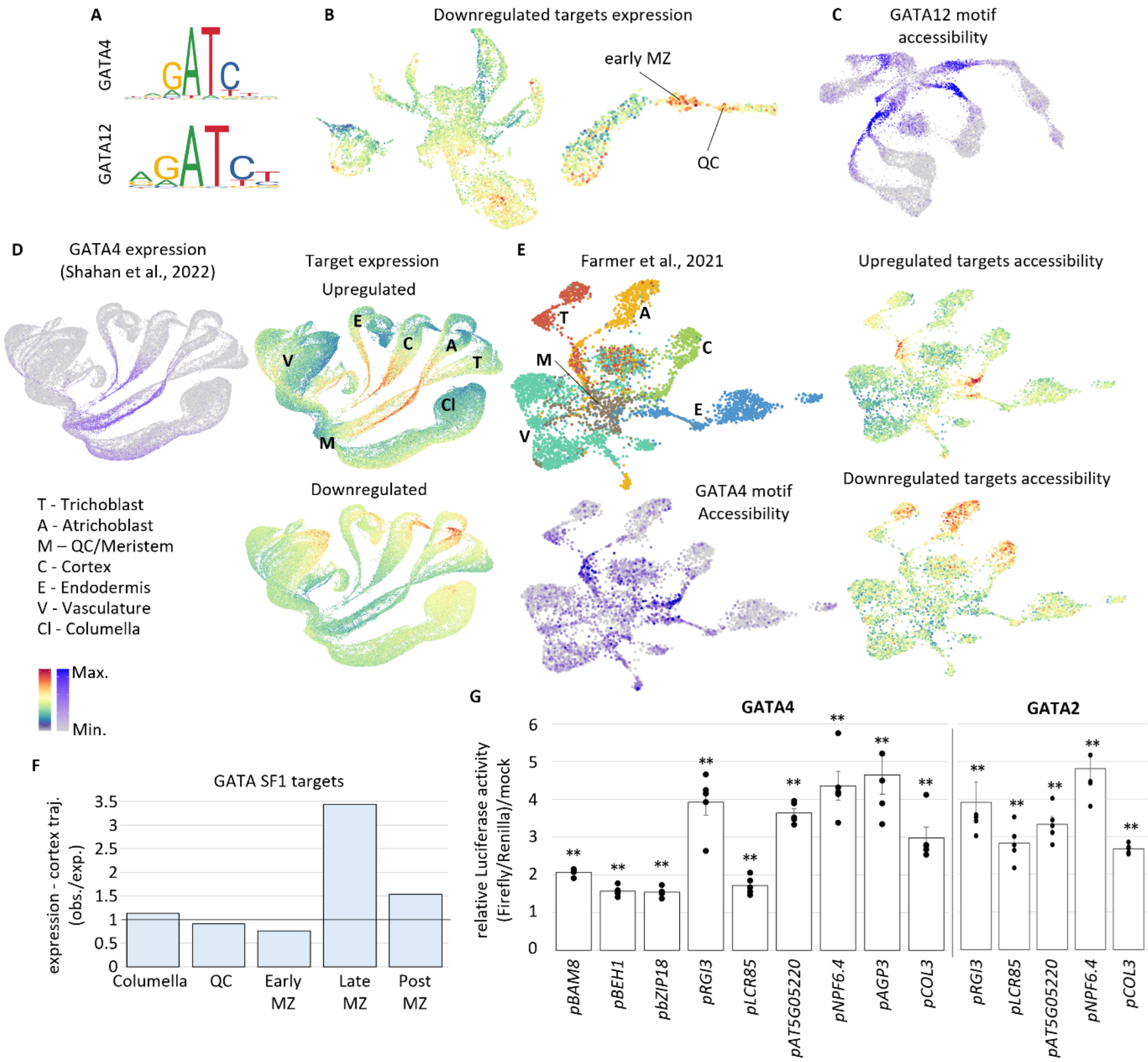

Supplementary Figure 4. SF1 GATA TFs determine root meristem size through opposing effects on genes linked to cell proliferation versus early differentiation (related to Figure 4). (A) Position Weight Matrices for the GATA4 and GATA12 DNA-binding motifs used in this study (Raulusevicíute et al., 2024). **(B)** UMAP showing enrichment of expression (module score) for predicted GATA SF1 targets, downregulated upon transient GATA2 or GATA4 expression (Supplemental Data 4) in the scRNA-seq atlas (left), and in the cortex-terminal trajectory (right). **(C)** Motif activity score illustrating accessibility of the GATA12 motif within the scATAC-Seq atlas **(D)** UMAPs showing expression of *GATA4* (top left), and enrichment of expression (module score) of GATA SF1 TF predicted targets upregulated (top right) or downregulated (bottom right) upon transient GATA4 expression in root culture protoplasts (Supplementary Data 4), within an independent scRNA-seq root atlas (Shahan et al., 2022) reinforce the observations. **(E)** Motif activity score illustrating accessibility of the GATA4 motif within an independent scATAC-Seq atlas (bottom left, Farmer et al., 2021), alongside enrichment of chromatin accessibility (module score) in promoters of predicted SF1 GATA targets upregulated (top right) or downregulated (bottom right) upon ectopic GATA4 expression in root culture protoplasts (Supplementary Data 4). Top left shows a minimal annotation of the atlas. **(F)** Enrichment plot illustrating that the predicted SF1 GATA targets that are differentially expressed along the cortex-terminal pseudotime trajectory (n=1,225) are preferentially expressed in the late- and post MZ, whereas the number of such genes expressed in the early MZ is below expectations, consistent with the lower percentage (22%) of predicted targets downregulated upon transient GATA2 or GATA4 expression. Data is plotted as the observed number of genes over the expected number based on the distribution of all DEGs in the trajectory (Supplementary Data 1,4). Black line: no change from expectation. **(G)** Relative luciferase activity

(mean ± se, n=5 biological replicates), calculated as Firefly over Renilla luciferase activity, shows significant upregulation of select GATA SF1 targets by GATA4 (left) and GATA2 (right) in Arabidopsis root culture protoplasts. Values shown are relative to mock controls (no TF). **p <0.01 two-sided Student's t-test. For replicate numbers and specific p values please see the Source Data file.

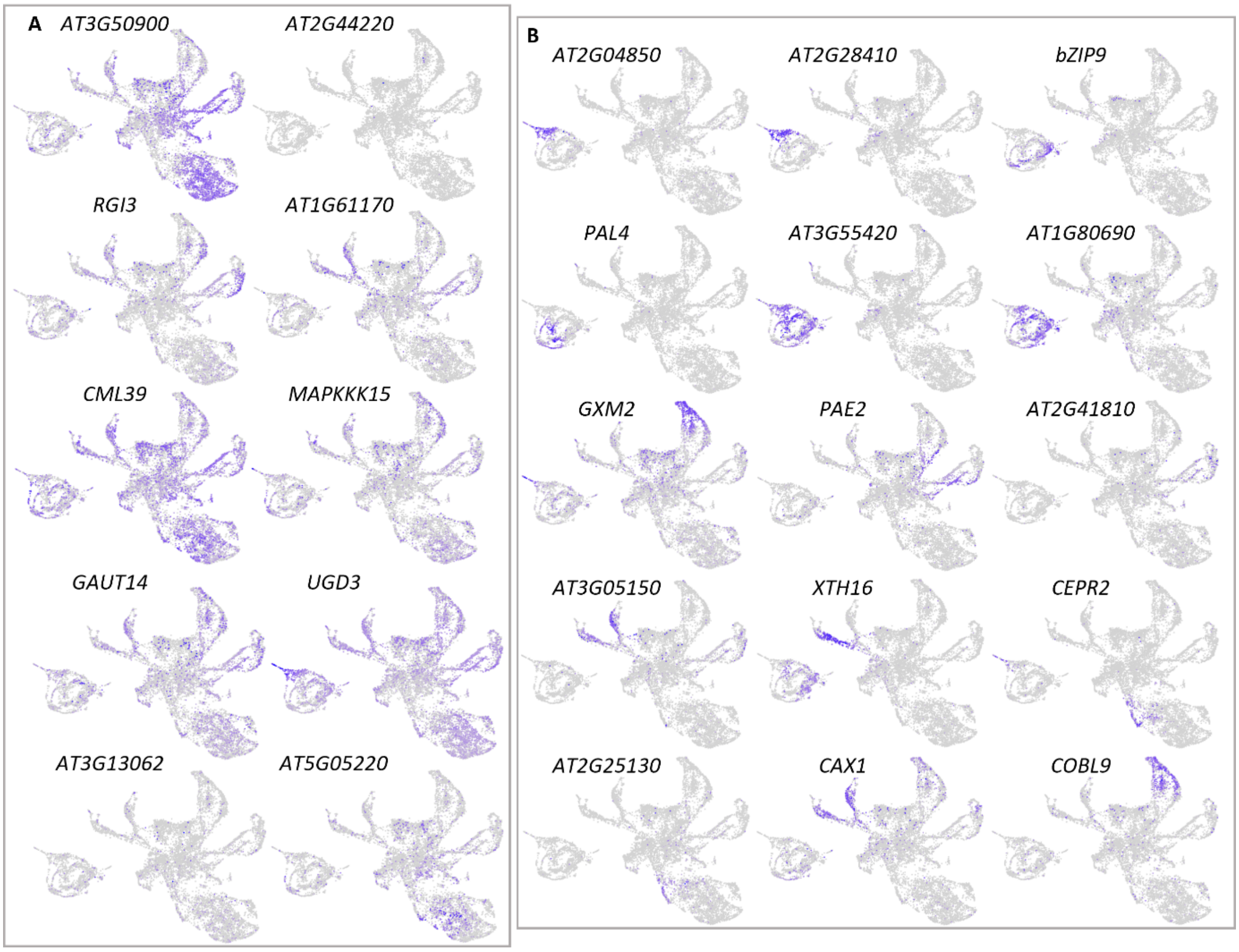

Supplementary Figure 5. A subset of SF1 GATA TF targets show a tissue specific pattern of expression (related to Figure 4). (A) UMAPs showing expression profiles in the scRNA-seq atlas for the predicted SF1 GATA targets examined in Fig. 4D. While most predicted targets show expression across multiple tissue layers in the root, a subset shows a varying degree of tissue specificity. **(B)** UMAPs showing tissue-specific expression profiles of additional select predicted SF1 GATA targets.

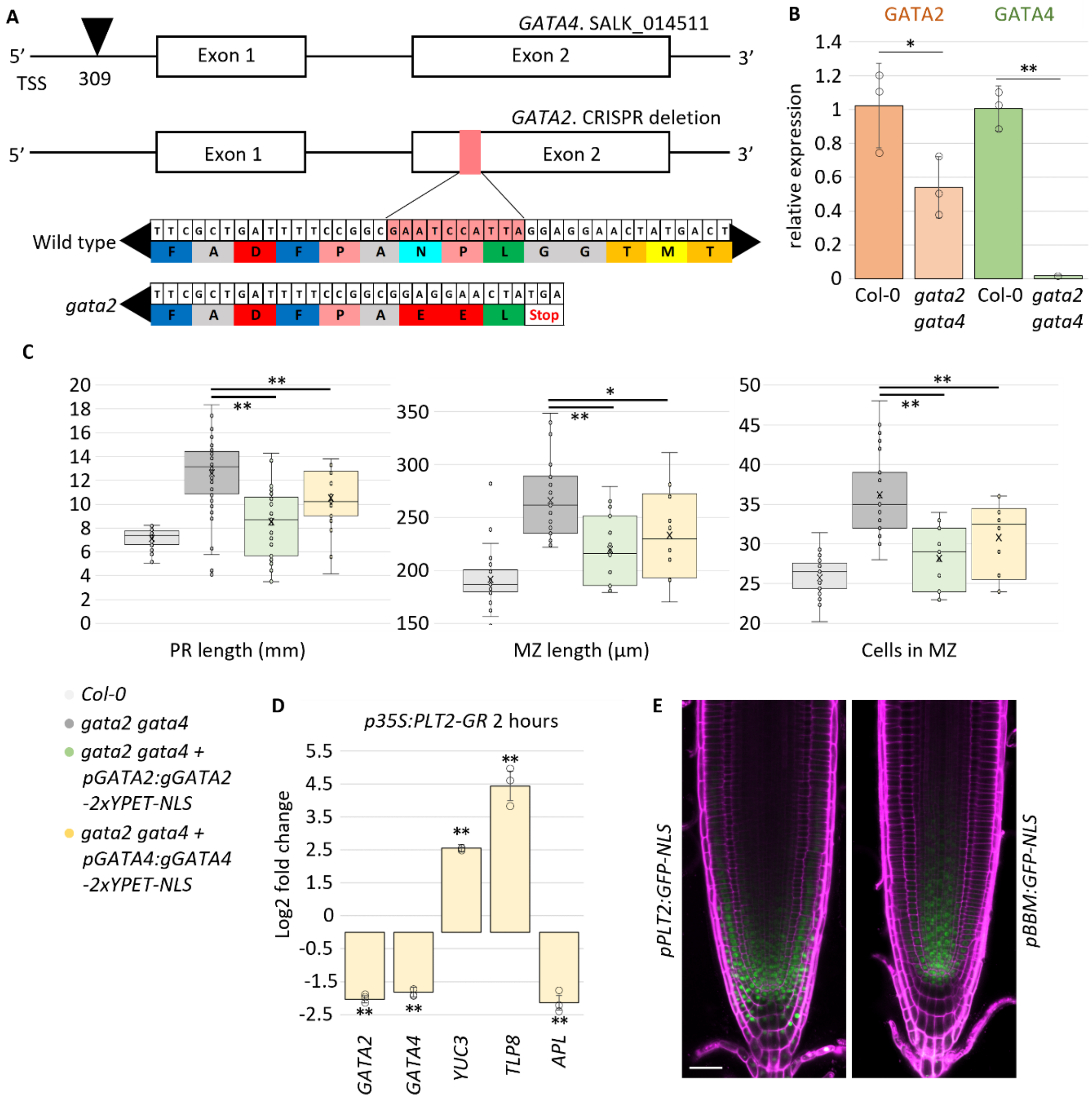

Supplementary Figure 6. SF1 GATA TFs act in a GRN with PLT to regulate root meristem size (related to Figures 4 and 5). (A) Schematics of the mutations in *gata2* and *gata4*. The *gata4* allele (SALK_014511) results from a T-DNA insertion in the 5′ UTR, ~309 bp from the GATA4 transcription start site (TSS), whereas the *gata2* allele carries a 10 bp deletion early in the second exon that generates a premature stop codon deleting nearly half the protein. **(B)** RT-qPCR analysis shows relative transcript levels (mean ± sd, n=3 biological replicates) of *GATA2* and *GATA4* are reduced ~40% and ~99%, respectively, in *gata2 gata4* versus wild-type Col-0. For each gene, one of the PCR primers spans the exon 1-exon 2 junction. **(C)** Box plots for primary root (PR) length, MZ length, and the number of cells in the MZ in 5-day old roots. These phenotypes are increased in *gata2gata4* and are variably recovered in T1 transgenic seedlings transformed with *pGATA2:gGATA2:2xYPET-NLS* or *pGATA4:gGATA4:2xYPET-NLS* in the *gata2gata4* background, illustrating functionality of the TF-fusions. **(D)** RT-qPCR analysis shows relative transcript levels (mean ± sd, n=4 biological replicates) for PLT2 targets decrease or increase significantly upon PLT2 induction (*p35S:PLT2-GR*, 2 hrs induction with 20 µM dexamethasone). Extension of Fig. 5A. **(E)** Expression of *PLT2* and *BBM* (*PLT4*) in the Arabidopsis root is limited to the SCN and early MZ. See also Galhina et al., 2007. Scale bars, 20 µm. *p <0.05, **p <0.01 two-sided Student's t-test. For replicate numbers and specific p values please see the Source Data file.
