# Supplementary material for: GATA transcription factors coordinate developmental progression in the arabidopsis root
Source: Nat Commun. 2026 Nov 3;17:11367. doi: 10.1038/s41467-026-78787-0 (PMC13731660; doi:10.1038/s41467-026-78787-0)
Supplement: Supplementary file 2 [file 41467_2026_78787_MOESM2_ESM.pdf]

Description of Additional Supplementary Files

Supplementary Data 1

Differentially expressed genes (DEGs) defining clusters of the Arabidopsis root scRNA-seq atlas, and zones along the pseudotime-derived cortex developmental trajectory.

Supplementary Data 2

All accessible genomic regions (ACRs) identified in the Arabidopsis root scATAC-seq atlas.
Differentially accessible chromatin regions (DARs) defining clusters in the scATAC-seq atlas.

Supplementary Data 3

DARs along a pseudotime-derived cortex developmental trajectory, with proximal genes and DEGs from the equivalent transcriptomic trajectory.

Supplementary Data 4

TF binding motif enrichment under DARs characterizing developmental zones along the pseudotime-derived cortex developmental trajectory. CellOracle defined GRNs. Positions of SF1 GATA binding motifs in ACRs in the scATAC-seq root atlas, and list of putative SF1 GATA targets. DEGs following transient GATA2 or GATA4 expression in root culture protoplasts.

Supplementary Data 5

Gene name abbreviations, gene references, and primers used in this study.
