# Supplementary material for: GATA transcription factors coordinate developmental progression in the arabidopsis root
Source: Nat Commun. 2026 Nov 3;17:11367. doi: 10.1038/s41467-026-78787-0 (PMC13731660; doi:10.1038/s41467-026-78787-0)
Supplement: Supplementary file 3 [file 41467_2026_78787_MOESM8_ESM.pdf]

nature portfolio

Peer Review File

GATA Transcription Factors Coordinate Developmental Progression in the Arabidopsis Root

Corresponding Author: Dr Marja Timmermans

This file contains all reviewer reports in order by version, followed by all author rebuttals in order by version.

Version 0:

Reviewer comments:

Reviewer #1

(Remarks to the Author)
Overall assessment
This is a valuable and well-executed study. By integrating single-cell transcriptomics, single-cell chromatin accessibility profiling, machine-learning-based network inference, and genetic and molecular experiments, the authors systematically investigate the transcriptional and chromatin regulatory dynamics underlying the transition from the meristematic zone to the elongation/differentiation zone in the Arabidopsis root. The manuscript provides a detailed characterization of the phased transition from proliferation to differentiation during ground tissue development and further identifies and validates the SF1-GATA transcription factors GATA2 and GATA4 as regulators of this developmental transition. Overall, the study is rich in data, carefully analyzed, and provides important insights into the temporal control of root development.
I consider this manuscript suitable for publication. Nevertheless, several key conclusions would benefit from further clarification or additional support, particularly regarding the annotation of scATAC-seq trajectory segments, the rationale for defining the late MZ as a regulatory hub, the relationship between GATA transcription factors and chromatin accessibility changes, and the interpretation of the auxin/CK feedback model. The following comments are intended to help improve the rigor of the argumentation and the clarity of figure presentation.

Major comments
1. Annotation of scATAC-seq trajectory segments relies heavily on label transfer and should be stated more cautiously or independently validated
A major conclusion of the manuscript is that cortex and endodermis diverge earlier at the chromatin accessibility level than at the transcriptomic level, and that this chromatin-level bifurcation occurs in the early MZ. However, the annotation of this "early MZ" region appears to rely mainly on label transfer from the scRNA-seq trajectory to the scATAC-seq trajectory. Since scATAC-seq itself reflects the relative topology of nuclei based on chromatin accessibility, but does not independently provide their precise spatial positions along the root axis, assigning the scATAC branch point specifically to the early MZ carries some uncertainty.
The manuscript notes that 278 DARs distinguish cortex and endodermis in the early MZ. This supports the presence of chromatin accessibility differences between these two populations within the label-transfer-defined early MZ, but it does not independently demonstrate that this region corresponds anatomically to the early MZ. I therefore suggest that the authors state the conclusion that "chromatin-level bifurcation occurs in the early MZ" more cautiously and explicitly acknowledge that this annotation mainly depends on cross-modality label transfer and currently lacks independent spatial validation.
If feasible, the authors should consider adding independent validation. For example, ATAC-seq after FACS isolation using spatial reporter lines, or reporter assays using representative early-MZ-associated DARs, could help test whether these accessible regions indeed show early-MZ-specific activity. At minimum, the authors should provide representative marker-associated accessible peaks in the main figures or supplementary figures to strengthen the confidence in the scATAC-seq regional annotation.

2. The rationale for defining the late MZ as a regulatory hub requires further support
The authors propose that the late MZ acts as a regulatory hub for the transition from cell proliferation to differentiation, and suggest that late-MZ DARs are associated with both early meristem/proliferation genes and later differentiation genes. However, the logic presented in Fig. 3A does not appear to uniquely support the specificity of the late MZ. Post-MZ DARs are also highly abundant and are similarly associated with a subset of early-expressed genes as well as differentiation-related genes. Therefore, based solely on the criterion that DAR-proximal genes span both early and late expression

phases, the post MZ could also be interpreted as a regulatory remodeling hub.
I suggest that the authors provide a more quantitative comparison of the regulatory features of late-MZ and post-MZ DARs, such as the proportions of DAR-associated genes linked to early proliferative genes versus later differentiation genes, enrichment levels, motif composition, and regulatory directionality. Such analyses would clarify why the late MZ, rather than the post MZ, should be considered the key regulatory hub.
In addition, Fig. 3A does not distinguish between DARs with increased accessibility and those with decreased accessibility. If the authors wish to support the model that the late MZ represses early meristem/proliferation genes while activating later differentiation genes, it would be important to separate increased-accessibility DARs from decreased-accessibility DARs. This would allow the authors to test whether decreased-accessibility DARs are preferentially associated with early meristem/proliferation genes, while increased-accessibility DARs are preferentially associated with later differentiation genes.

3. The link between GATA transcription factors and chromatin accessibility analysis needs to be better integrated
A major strength of the first part of the manuscript is the integration of scRNA-seq and scATAC-seq, leading to the conclusion that the late/post-MZ transition is accompanied by substantial chromatin accessibility remodeling, which is used to explain the regulatory basis of the transition from proliferation to differentiation. However, the subsequent functional validation of GATA2/GATA4 mainly focuses on transcriptional regulation and developmental phenotypes, including reporter analyses, transient expression RNA-seq, inducible overexpression, mutant phenotyping, and RT-qPCR. These data strongly support a role for GATA2/GATA4 in the proliferation-to-differentiation transition, but they do not directly establish how GATA2/GATA4 are connected to the chromatin accessibility changes described earlier.
As a result, the current narrative appears somewhat disconnected: the first part emphasizes chromatin accessibility remodeling as a key feature of the late-MZ transition, whereas the second part mainly demonstrates that GATA2/GATA4 regulate transcriptional programs and meristem size. The authors should clarify in the Results and Discussion whether GATA2/GATA4 are proposed to act as upstream drivers of chromatin remodeling, or primarily as transcriptional regulators acting within a developmental transition that is accompanied by chromatin remodeling.
If the authors favor the former interpretation, namely that GATA2/GATA4 directly participate in chromatin opening or closing in the late MZ, more direct evidence would be needed, such as ATAC-seq or CUT&Tag after GATA perturbation. If such evidence is not currently available, the authors should reduce causal wording in the model and text, and more cautiously state that GATA2/GATA4 regulate the transcriptional switch associated with chromatin remodeling, rather than implying that GATA2/GATA4 have been demonstrated to directly drive chromatin remodeling.

4. The relationship between ARR-B and PLT in Fig. 5A and Fig. 5E requires further clarification
Fig. 5A shows that, after p35S:GATA4-GR induction, PLT/BBM genes exhibit a transient or biphasic response: they are repressed at early time points but increase again after 4 h. At the same time, ARR1 and ARR12 are upregulated over the induction time course. However, in the model shown in Fig. 5E, ARR-B factors are described as repressors of PLT or the auxin-PLT program. Thus, the simultaneous increase of ARR1/ARR12 and PLT genes at 4 h is not fully consistent with the simple model in which ARR-B represses PLT.
The authors interpret the 4 h increase in PLT genes as being related to ARR-B/auxin feedback. However, Fig. 5A does not provide key intermediate evidence for this feedback mechanism. For example, if PLT recovery is caused by feedback, the authors should show whether auxin signaling or auxin-related genes recover or increase at 4 h, whether GATA2/GATA4 or GATA downstream activity changes accordingly, and whether the increase in PLT transcripts corresponds to recovery of PLT protein patterns or PLT activity. Without such evidence, the current data demonstrate only that GATA4-GR induction causes a biphasic transcriptional response of PLT genes, but do not directly establish that this response is driven by ARR-B/auxin feedback.
I suggest that the authors provide additional data, such as auxin reporter dynamics, auxin-related gene expression, GATA target gene dynamics, or PLT protein reporters, to support this feedback interpretation. If such experiments are not feasible at this stage, the authors should revise the text and the Fig. 5E model to more clearly distinguish early GATA4-induced PLT repression from later secondary or compensatory responses, and should avoid directly attributing the 4 h PLT increase to ARR-B/auxin feedback without additional support.

Minor comments
5. Fig. 1A and Fig. 2A
The UMAPs in Fig. 1A and Fig. 2A would benefit from clearer cell-type or tissue-region annotations. Since the downstream analyses rely heavily on the identification of SCN, QC, columella, cortex, and endodermis populations, direct labeling in the main figures would greatly improve readability.
6. Fig. 1E and the rationale for GATA4 inclusion later in the manuscript
Fig. 1E lists marker genes used to define different developmental phases. Since GATA4 is later analyzed as a late-MZ-associated SF1-GATA factor, the authors should explain why GATA2, but not GATA4, was included in Fig. 1E. If GATA4 is less restricted to the late MZ than GATA2, this should also be more clearly discussed when GATA4 is introduced as a late-MZ regulator.
7. Fig. 2B
Some labels in Fig. 2B appear to be corrupted and should be corrected.
8. GATA2/GATA4 expression patterns should be shown before Fig. 3C
Before presenting the CellOracle perturbation predictions in Fig. 3C, it would be helpful to directly show the expression patterns of GATA2 and GATA4 in the relevant scRNA clusters or along the cortex trajectory, for example using FeaturePlots, trajectory expression plots, or module scores.
9. The apparent inconsistency between GATA expression timing and predicted perturbation timing requires clarification
Based on the reporter images in Fig. 3D/E, GATA4 appears to initiate later and to extend more broadly into the post MZ, whereas GATA2 appears to initiate earlier and to be more spatially restricted. However, in the CellOracle perturbation

analysis shown in Fig. 3C, the predicted effect of GATA4 loss appears to occur at an earlier developmental stage than that of GATA2. This is not immediately intuitive, because one might expect the perturbation timing to broadly correspond to the spatial-temporal expression domains of the respective TFs. Additional evidence or robustness analysis would be helpful to support the reliability of the differential perturbation patterns predicted for GATA2 and GATA4.
10. The GATA2 reporter construct should be labeled consistently in Fig. 1M and Fig. 3D
Fig. 1M and the left panel of Fig. 3D appear to show the same type of GATA2 promoter reporter construct, but the construct names are labeled differently. Fig. 1M is labeled as pGATA2::2xYPET, whereas Fig. 3D is labeled as pGATA2::2xYPET-NLS. If the same construct was used, the construct name and figure legend should be made consistent to avoid confusion.
11. The earlier onset of GATA2 expression in the epidermis than in GT should be discussed
In Fig. 1M and Fig. 3D, GATA2 expression appears to initiate earlier in the epidermis than in the GT/cortex. Since the manuscript mainly focuses on the GT trajectory, the authors should clarify whether SF1-GATA factors are proposed to coordinate the proliferation-to-differentiation transition across multiple tissues, or whether their timing and function differ between the epidermis and GT. If the former is the case, the apparent difference in the onset of GATA expression between epidermis and GT raises the question of whether the MZ/EZ transition is asynchronous across different tissues. This point would be worth briefly discussing.

Reviewer #2

(Remarks to the Author)
The publication discusses the role of GATA transcription factors in coordinating developmental progression in plants. The paper is significant because it uses single-cell transcriptomic data to provide deep functional insights into the process. There have been significant publications on the GATA transcription factor in recent years, including a paper by Kim (2024) in Nature. However, most publications to date have focused on the expression patterns of the GATA transcription factors. To my knowledge, the work discussed in this publication is novel and provides significant insight.
The methodology is sound, in that the initial in silico analysis is followed by promoter fusion analysis and transient expression using protoplast. These results are further confirmed by GATA2 knockout mutant phenotypes. The inclusion of the mutant analysis is a strong positive point for the paper. In addition, the use of mutants and p35S:GATA4-GR lines shows that the authors conducted a strong genetics-based analysis to confirm the initial in silico findings. The methodology is sufficient for reproducibility.

In conclusion, I believe that this manuscript presents a thorough analysis of a novel aspect of the GATA transcription factors and oragan development in general. I recommend it for publication.

Reviewer #3

(Remarks to the Author)
The manuscript by Denyer et al. investigates root developmental progression in the Arabidopsis root ground tissue. By integrating scRNA-seq and scATAC-seq datasets, the authors identified a major transcriptional switch occurring in the late meristematic zone that mediates the differentiation of endodermis and cortex cells. Further analyses pinpointed and experimentally validated GATA transcription factors as key regulators of this transcriptional transition. Overall, this study reveals an exciting mechanism that coordinates cell proliferation and differentiation during root development. However, several issues need to be addressed before the conclusions can be fully supported.

Major issues:

1. The study relies primarily on a single scRNA-seq dataset (Dataset S1, Fig. 1A) for downstream analyses. Since several additional Arabidopsis root scRNA-seq datasets have been published previously, it would be important to determine whether the identified developmental trajectories and transcriptional programs are reproducible across independent datasets. Additionally, because this study represents a re-analysis of a previously published dataset (Denyer et al., 2019), a brief description of the dataset, including the number of cells, sequencing platform, and major processing steps, should be provided. The marker genes used for cell cluster annotation should also be listed. Furthermore, Dataset S1 is not sufficiently self-explanatory and is difficult to interpret. Without access to the original Seurat RData objects, the information provided in the table is limited. Additional annotation information and explanations should be included to improve its usability.

2. The authors define five expression phases along the developmental trajectory in Figure 1B and 1C: columella, QC, Early MZ, Late MZ, and Post MZ. This classification may be confusing because QC represents the starting point of both the columella lineage toward the root tip and the meristematic zone lineage toward the shootward direction. A clearer organization would be to define QC as the initial developmental stage, with columella representing one differentiation trajectory, while Early MZ, Late MZ, and Post MZ represent subsequent developmental phases (e.g., phases 2, 3, and 4) along the ground tissue lineage.

3. GATA transcription factors constitute a large gene family, and functional redundancy among family members is likely. Therefore, the expression patterns of all GATA family members should be examined to determine whether GATA2 displays a unique expression pattern or whether other GATA genes show similar expression dynamics during root development.

4. In Figure 2A and Figure S3C, the criteria used to select cells for trajectory analysis are unclear. Compared with the scRNA-seq UMAP, additional cell groups appear at the terminal branches of cortex and endodermis trajectories but were not included in the trajectory analysis. The authors should clarify how cells were selected and explain why these

populations were excluded.

5. In Lines 204–205, the authors state that “more DEGs proximal to post-MZ DARs are expressed preferentially in the QC and early MZ.” This statement requires clarification. Does this indicate that the corresponding chromatin regions are inaccessible in QC and early MZ but become accessible in post-MZ cells? However, several of these genes, such as WOX5 and TMO7, are already expressed in QC or early MZ cells, which appears contradictory. The relationship between chromatin accessibility dynamics and gene expression patterns should therefore be clarified.

6. In Figure 3B, although several motifs show increased activity during the late MZ stage, GATA1, GATA4, and GATA12 motifs are not among the most strongly enriched motifs, and their enrichment decreases in the post-MZ stage. How do the authors reconcile this transient and intermediate enrichment pattern with the proposed central role of GATA factors in controlling the developmental transition?

7. In Figure 4D, the authors examine MZ cell numbers in the gata2 gata4 double mutant. It would be important to discuss whether other GATA family members are still expressed in the MZ region and whether they could partially compensate for the loss of GATA2 and GATA4 functions.

8. In Lines 354–394, the authors describe an interaction network between GATA factors and auxin signaling components (PLTs) as well as cytokinin signaling components (ARRs). However, whether these interactions are functionally important for root development remains largely untested. Therefore, the section title, “GATA TFs govern developmental progressions in a network integrating auxin and cytokinin signaling,” may overstate the conclusions. A more appropriate title reflecting the observed associations rather than direct functional integration would be preferable.

Minor issues:

1. In Figure 1, it is unclear which clusters in the UMAP (Figure 1A) correspond to columella, QC, endodermis, and cortex populations. It would be helpful to provide a supplemental figure showing Figure 1B using the same color scheme as Figure 1A, allowing readers to easily understand which cell populations were selected for trajectory analysis.

2. In Figure 1C, the criteria used to define QC, Early MZ, Late MZ, and Post MZ stages should be clarified. The y-axis of Figure 1C is also confusing. Does it represent groups or modules of marker genes associated with each developmental stage? Similarly, in Figures S2B and S2C, representative gene names should be provided to facilitate interpretation.

3. The expression patterns of selected marker genes, particularly those associated with branch points of the trajectory, should also be visualized on the UMAP shown in Figure 1B.

4. In Figure 2B, the authors analyze 500–1000 bp selected regions together with 2.5 kb gene body regions. The rationale for selecting these regions is unclear. Why were promoter regions not included, considering that TF binding motifs are often located within promoter regions? Additionally, in Figure 3A, the criteria used to define “2.5 kb region-specific DARs” should be clearly explained.

5. In Lines 363–375, GATA factors appear to activate ARR expression while repressing PLT expression. The authors should discuss the dual regulatory nature of transcription factors, which can function as both activators and repressors depending on genomic context and interacting partners.

Reviewer #4

(Remarks to the Author)
The submitted manuscript investigates the role of GATA transcription factors (TF) during cell differentiation and fate acquisition along Arabidopsis root tip, using a mix of scRNA-seq, transgenic lines, protoplast assay and microscopy approaches.

The authors have exploited published scRNA-seq datasets, focusing on analysis of the cortex/endodermis cell fate split as the two cell lineage derive from a single common initial cell and appear well identified in the scRNA-seq datasets.

They generated lines expressing selected cell identity markers and lines co-expressing confocal markers for cell identities and GATA expression to show the spatio-temporal sequence of genes expression in the root apex and in endodermis cells.

The authors also mapped chromatin accessibility and created an atlas of accessible chromatin regions, highlighting the overlap between clusters of chromatin accessibility profiles and clusters according to cell identities from scRNA-seq data. They focused on the dynamics of gene expression the cortical-terminal cells to identify the GATA TF family as potential master regulators for the identity acquisition in this lineage, through a combination of TF binding motifs analysis, gene network analysis and in silico modelling with CellOracle. The implication of GATA TF in the regulation of cell identity acquisition processes is further tested in vitro through protoplastic RNA-seq analysis and luciferase assay for putative GATA targets and in vivo through confocal observation of identity markers in gata double mutant background.

The study in itself is well built and very comprehensive and the results are impressive. I nevertheless have a few major questions and some minors remarks.

Major Reflexions :
- if the dynamics of gene expression are so similar in the two cell lineage considered (cortex/endodermis) until the very late MZ - what causes the switch to one or the other cell fate ? Is it linked with the fact that the endodermis layer is closer to the stele and receive additional positional information (hormone gradient ?) which would send it towards its specific fate compared to cortex ? This particular point warrant more discussion from the authors, especially so since they propose a model of progression through proliferation/differentiation regulated by the auxin/cytokinin balance, which is know to be implicated in radial patterning of the root as well and mechanisms such as lateral root organogenesis and patterning.

- in the gata2 gata4 mutant the MZ appear longer and holds more cells, indicating that the differentiation process seems to take longer than in WT - but it ultimately occurs despite the absence of GATA. This suggests an alternative way to regulate the cell differentiation, albeit slower than under GATA control, and the authors suggests potential GATA12 redundancy to explain the still completing differentiation course. Ideally, the phenotype of gata12 mutant and double/triple gata 2/4/12 should be investigated to corroborate the conclusion that the GATA family is the linchpin of the shift between proliferation and differentiation. The definitive proof of this assertion would be for the differentiation to be blocked altogether, unable to progress in absentio of GATA TFs. Otherwise, the conclusion should be toned down.

- The model presented in fig 5E is incoherent with the data from panel 5A : in the proposed negative feedback loop, there is no possibility for the observed resurgence of PLT expression. A potential compatible explanation would rather be that a gene downstream of GATA or ARR would instead promote the delayed PLT expression resurgence, providing retrocontrol on the whole regulatory cascade. The proposed model should include this to be coherent with the data supporting it.

Minor Questions & Remarks :

- Can the authors justify why they used the TAIR10 and 10.55 reference for their analysis, when the version 12 is available since February 2026 ?

- L21 "where RNA...coordination" - please reformulate. While plants lack promoter-proximal pausing seen in metazoans, they exhibit other forms of Pol II regulation such as 3' Pol II pausing (e.g. https://doi.org/10.1038/s41467-019-12328-w), so 'not well established' is an overstatement.

- L44 "Realizes" - unproper term, prefer the use of "induces"

- L46-47 "although...analogy" - Please remove. One can argue that the state of cell proliferation exists as an uphill part of the landscape, present as a transient state (i.e. a local minimum of altitude like a small indent in the landscape) of gene expression corresponding to a proliferating state, from which the cells can be pushed out to then go downhill to a true 'minimum' corresponding to a differentiated state.

- fig 4 legend : (B) mention 'above' and 'below' subpanel image positionning - there is only left and right subpanel images ? please correct.

- figS2 legend : "progress left and rich" -> "left and right" / "top right is a heatmap (as in 1E) -> the heatmap is not in the top-right position.

- figS2, panel D : please precise the zones denomination (early/late MZ/post MZ) on the pictures.

- figS2, panel D : could the authors provide complementary information on the CHERRY tagged protein stability, as while the expression of GATA2 appears transient in the provided heatmap, the expressed marked protein under pGATA2 is present in the cortex lineage past late MZ boundary as attested by RGl3 and AT1G62500 promoter activities ?

- minor typos : legends of multiple data sheets for supp dataset S1 "DEG were dientified" -> "identified"

- minor typos : supp dataset S3 legends for multiple data sheets "accesible regions" -> "accessible" / "cortex tajectory" -> "trajectory" / "change in accesibility" -> "accessibility" / "adjted p-value" -> "adjusted" / "cortex-terminal trajecotry" -> "trajectory" / "seperated by developemental region" -> "separated"

- minor typos : supp dataset S4 legends for multiple data sheets "seperated by developmental region" -> "separated" / "trasnient expression" -> "transient"

Version 1:

Reviewer comments:

Reviewer #1

(Remarks to the Author)
All my concerns have been addressed or clarified. Therefore, I am happy to recommend it for publication in nat comms.

Reviewer #3

(Remarks to the Author)
The authors have thoroughly addressed all prior concerns.

Reviewer #4

(Remarks to the Author)
I thanks the authors for their responses to my comments and to the other reviewers'. The revised manuscript is improved and satisfactory. My main remaining concern is still the model in figure 5E which has not been updated or nuanced to be coherent with the data and should be in my personal opinion considering its importance in the manuscript.

I leave the final appreciation of this point to the editor, and have no further comments at that stage.

Open Access This Peer Review File is licensed under a Creative Commons Attribution 4.0 International License, which permits use, sharing, adaptation, distribution and reproduction in any medium or format, as long as you give appropriate credit to the original author(s) and the source, provide a link to the Creative Commons license, and indicate if changes were made.
In cases where reviewers are anonymous, credit should be given to 'Anonymous Referee' and the source.
The images or other third party material in this Peer Review File are included in the article's Creative Commons license, unless indicated otherwise in a credit line to the material. If material is not included in the article's Creative Commons license and your intended use is not permitted by statutory regulation or exceeds the permitted use, you will need to obtain permission directly from the copyright holder.
To view a copy of this license, visit https://creativecommons.org/licenses/by/4.0/

Reviewer #1 (Remarks to the Author)

Overall assessment

This is a valuable and well-executed study. By integrating single-cell transcriptomics, single-cell chromatin accessibility profiling, machine-learning-based network inference, and genetic and molecular experiments, the authors systematically investigate the transcriptional and chromatin regulatory dynamics underlying the transition from the meristematic zone to the elongation/differentiation zone in the Arabidopsis root. The manuscript provides a detailed characterization of the phased transition from proliferation to differentiation during ground tissue development and further identifies and validates the SF1-GATA transcription factors GATA2 and GATA4 as regulators of this developmental transition. Overall, the study is rich in data, carefully analyzed, and provides important insights into the temporal control of root development.

I consider this manuscript suitable for publication. Nevertheless, several key conclusions would benefit from further clarification or additional support, particularly regarding the annotation of scATAC-seq trajectory segments, the rationale for defining the late MZ as a regulatory hub, the relationship between GATA transcription factors and chromatin accessibility changes, and the interpretation of the auxin/CK feedback model. The following comments are intended to help improve the rigor of the argumentation and the clarity of figure presentation.

Major comments

1. Annotation of scATAC-seq trajectory segments relies heavily on label transfer and should be stated more cautiously or independently validated. A major conclusion of the manuscript is that cortex and endodermis diverge earlier at the chromatin accessibility level than at the transcriptomic level, and that this chromatin-level bifurcation occurs in the early MZ. However, the annotation of this "early MZ" region appears to rely mainly on label transfer from the scRNA-seq trajectory to the scATAC-seq trajectory. Since scATAC-seq itself reflects the relative topology of nuclei based on chromatin accessibility, but does not independently provide their precise spatial positions along the root axis, assigning the scATAC branch point specifically to the early MZ carries some uncertainty.

The manuscript notes that 278 DARs distinguish cortex and endodermis in the early MZ. This supports the presence of chromatin accessibility differences between these two populations within the label-transfer-defined early MZ, but it does not independently demonstrate that this region corresponds anatomically to the early MZ. I therefore suggest that the authors state the conclusion that "chromatin-level bifurcation occurs in the early MZ" more cautiously and explicitly acknowledge that this annotation mainly depends on cross-modality label transfer and currently lacks independent spatial validation.

If feasible, the authors should consider adding independent validation. For example, ATAC-seq after FACS isolation using spatial reporter lines, or reporter assays using representative early-MZ-associated DARs, could help test whether these accessible regions indeed show early-MZ-specific activity. At minimum, the authors should provide representative marker-associated accessible peaks in the main figures or supplementary figures to strengthen the confidence in the scATAC-seq regional annotation.

Label transfer informed by RNA-seq is considered a robust method for annotating scATAC-seq data, and is broadly and successfully used as such (see Dorrity et al 2021; Farmer et al 2021;

Zhang et al., 2023; Sun et al., 2024, for example). This point is also evident through a comprehensive comparison of various methods (see Fu et al., 2025 for a benchmarking study comparing methodologies). However, we understand the reviewer's comment and have now also employed GLUE (graph-linked unified embedding) as a complimentary approach to annotate the ground tissue trajectory (Cao and Gao, 2022). This method is highly regarded in the aforementioned benchmarking study, and uses a distinct methodology. The Seurat integration pipeline converts the ATAC data into a shared gene-level feature space and projects query cells onto the reference to predict labels. GLUE instead learns an unsupervised joint embedding space through a graph neural network that explicitly links chromatin accessibility and gene expression features. Importantly, the results support the earlier Seurat-based Label transfer annotation (Fig. S3D, new Fig S3E). Accurate annotation of the trajectory is further reflected in the prediction of GATA TFs as key regulators in the late MZ, and the support of this by experimental data. To further support the annotation, we now also provide a new supplementary figure illustrating differential chromatin accessibility at marker genes along the trajectory (Figs. 1E, S3F).

On both transcriptomic and chromatin levels, the boundary between the QC and the early MZ is clearest defined, as is evident from the heatmaps (Figs 1C, 2D), reporter gene expression patterns (Fig. 1E), and expression profiles of genes associated with the early MZ DARs along the cortex-terminal trajectory (Fig. 3A). The bifurcation in the chromatin trajectory occurs close to this "border", giving us great confidence that this happens at the beginning of the early MZ, long before the clear late MZ division of the RNA-seq trajectory. It is true that we cannot pinpoint its position on a cell-to-cell level, but it follows that the branchpoint in the chromatin trajectory occurs around the point at which the initial divides. We have modified the language around this aspect, as suggested.

2. The rationale for defining the late MZ as a regulatory hub requires further support. The authors propose that the late MZ acts as a regulatory hub for the transition from cell proliferation to differentiation, and suggest that late-MZ DARs are associated with both early meristem/proliferation genes and later differentiation genes. However, the logic presented in Fig. 3A does not appear to uniquely support the specificity of the late MZ. Post-MZ DARs are also highly abundant and are similarly associated with a subset of early-expressed genes as well as differentiation-related genes. Therefore, based solely on the criterion that DAR-proximal genes span both early and late expression phases, the post MZ could also be interpreted as a regulatory remodelling hub.

I suggest that the authors provide a more quantitative comparison of the regulatory features of late-MZ and post-MZ DARs, such as the proportions of DAR-associated genes linked to early proliferative genes versus later differentiation genes, enrichment levels, motif composition, and regulatory directionality. Such analyses would clarify why the late MZ, rather than the post MZ, should be considered the key regulatory hub. In addition, Fig. 3A does not distinguish between DARs with increased accessibility and those with decreased accessibility. If the authors wish to support the model that the late MZ represses early meristem/proliferation genes while activating later differentiation genes, it would be important to separate increased-accessibility DARs from decreased-accessibility DARs. This would allow the authors to test whether decreased-accessibility DARs are preferentially associated with early meristem/proliferation genes, while increased-accessibility DARs are preferentially associated with later differentiation genes.

The most important reason for why we propose the late MZ as a 'regulatory hub' is that the switch in gene expression programs underlying the branch point in the GT RNA-seq trajectory maps here (Fig. 1C, Dataset S1). Secondly, whereas few DEGs are prominent in the late MZ (Fig. 1C, Dataset

S1), a disproportionate high number of chromatin changes occur in these cells (Fig. 2D). Thirdly, the position of the late MZ of the root, between a region of rapidly dividing cells and cells commencing elongation/differentiation, makes the chromatin dynamics and regulatory action within of particularly interest. Fourthly, regarding the proportions mentioned by the reviewer, ~55.5% of DEGs proximal to late MZ-DARs are expressed in the QC or early MZ (see Lines 245-247). For the post MZ DARs, this proportion is considerably lower. Post MZ DARs are prominently associated with DEGs expressed in the same zone. The percentage of QC or early MZ-expressing genes proximal to post MZ DARs is ~31%, and this is likely an overestimation. Nearly 50% of Post MZ DARs are associated with genes expressed further up the root (see lines 219, 223), in cells not represented in the RNA-seq trajectory, perhaps as a consequence of nuclei capture being unrestricted by size, unlike for cells. The reviewer, therefore, is correct that there is also considerable feedback on the MZ from the post MZ, although we do not explore this in much detail so as not to dilute the story. We agree with the reviewer that we could have clarified these points better, and have now done so in the revised manuscript.

Regarding the reviewer's point on Figure 3A, these heatmaps only show genes associated with increased accessibility, and we now clarify this in the figure legend. However, increased accessibility does not suggest exclusively positive action on genes, as TFs often act as repressors on nearby genes, as reflected by late- and post-MZ DARs associated with genes expressed in the QC and early MZ (see also Figures 4 and S4). We do understand the reviewer's interest in chromatin regions that close at points along the trajectory, as these form a valuable lead into epigenetic regulation of developmental gene expression, but feel that given the wealth of information already presented in the manuscript, these would fall outside the current scope.

3. The link between GATA transcription factors and chromatin accessibility analysis needs to be better integrated. A major strength of the first part of the manuscript is the integration of scRNA-seq and scATAC-seq, leading to the conclusion that the late/post-MZ transition is accompanied by substantial chromatin accessibility remodeling, which is used to explain the regulatory basis of the transition from proliferation to differentiation. However, the subsequent functional validation of GATA2/GATA4 mainly focuses on transcriptional regulation and developmental phenotypes, including reporter analyses, transient expression RNA-seq, inducible overexpression, mutant phenotyping, and RT-qPCR. These data strongly support a role for GATA2/GATA4 in the proliferation-to-differentiation transition, but they do not directly establish how GATA2/GATA4 are connected to the chromatin accessibility changes described earlier.
As a result, the current narrative appears somewhat disconnected: the first part emphasizes chromatin accessibility remodeling as a key feature of the late-MZ transition, whereas the second part mainly demonstrates that GATA2/GATA4 regulate transcriptional programs and meristem size. The authors should clarify in the Results and Discussion whether GATA2/GATA4 are proposed to act as upstream drivers of chromatin remodeling, or primarily as transcriptional regulators acting within a developmental transition that is accompanied by chromatin remodeling.
If the authors favor the former interpretation, namely that GATA2/GATA4 directly participate in chromatin opening or closing in the late MZ, more direct evidence would be needed, such as ATAC-seq or CUT&Tag after GATA perturbation. If such evidence is not currently available, the authors should reduce causal wording in the model and text, and more cautiously state that GATA2/GATA4 regulate the transcriptional switch associated with chromatin remodeling, rather than implying that GATA2/GATA4 have been demonstrated to directly drive chromatin remodeling.

The nature of the GATA2 and GATA4 targets, as well as the phenotypes observed in gata2 gata4 and upon induced GATA4 overexpression support a role for these TFs in regulating the transition

from cell proliferation to differentiation. However, we do not have evidence as to whether the GATA TFs are the cause or a consequence of the chromatin accessibility changes. GATAs have been shown to remodel chromatin in mammalian systems (May et al., 2013; Gharui and Sengupta, 2025). However, to convincingly demonstrate potential pioneer transcription factor activity, for example, would necessitate a challenging and highly involved study on its own, one that we feel is well beyond the remit of this paper. We have clarified in the revised manuscript that we cannot distinguish at this stage whether the GATA TFs contribute directly to chromatin remodeling.

The two halves of the paper are connected in that we first describe the transcriptional dynamics underlying GT development, identifying a switch in gene expression connected to the transition from cell proliferation to cell differentiation. This switch in gene expression is a new distinguishing feature of the late MZ. As such, we used scATAC-seq, CellOracle, and TF binding motif enrichment analysis to identify associated changes in chromatin accessibility and pinpoint candidate transcriptional regulators underlying this switch. In these regards the analyses are highly informative, pointing to a prominent role for the SF1 GATA TFs. To avoid the work branching out in too many directions, we decided to focus on verifying contributions from the SF1 GATA TFs to the gene expression switch in the late MZ, rather than presenting additional global analyses. We believe that this makes for a stronger piece of work that demonstrates strategies to explore the wealth of information in single cell data and discern mechanistic insights from it. Since we are aware of the richness of data captured within scRNA-seq and scATAC-seq atlases and the potential for further data mining, we encourage the community to use both resources for varied projects by providing fully processed RDS objects as well as an intuitive online data browser.

4. The relationship between ARR-B and PLT in Fig. 5A and Fig. 5E requires further clarification Fig. 5A shows that, after p35S:GATA4-GR induction, PLT/BBM genes exhibit a transient or biphasic response: they are repressed at early time points but increase again after 4 h. At the same time, ARR1 and ARR12 are upregulated over the induction time course. However, in the model shown in Fig. 5E, ARR-B factors are described as repressors of PLT or the auxin-PLT program. Thus, the simultaneous increase of ARR1/ARR12 and PLT genes at 4 h is not fully consistent with the simple model in which ARR-B represses PLT.
The authors interpret the 4 h increase in PLT genes as being related to ARR-B/auxin feedback. However, Fig. 5A does not provide key intermediate evidence for this feedback mechanism. For example, if PLT recovery is caused by feedback, the authors should show whether auxin signaling or auxin-related genes recover or increase at 4 h, whether GATA2/GATA4 or GATA downstream activity changes accordingly, and whether the increase in PLT transcripts corresponds to recovery of PLT protein patterns or PLT activity. Without such evidence, the current data demonstrate only that GATA4-GR induction causes a biphasic transcriptional response of PLT genes, but do not directly establish that this response is driven by ARR-B/auxin feedback.
I suggest that the authors provide additional data, such as auxin reporter dynamics, auxin-related gene expression, GATA target gene dynamics, or PLT protein reporters, to support this feedback interpretation. If such experiments are not feasible at this stage, the authors should revise the text and the Fig. 5E model to more clearly distinguish early GATA4-induced PLT repression from later secondary or compensatory responses, and should avoid directly attributing the 4 h PLT increase to ARR-B/auxin feedback without additional support.

We understand the concern of the reviewer. Feedback between the PLT and ARR-B TFs, cytokinin and auxin is complex. This is perhaps best illustrated by the collapsed root meristem phenotype of *arr1arr10arr12* mutants, which is counterintuitive with the role of the ARRs in promoting cell elongation/differentiation, and indeed, conflicts with the extended meristem phenotype of

arr1arr12 (Dello Ioio et al., 2007; Argyros et al., 2008). To leave open the specific nature of the feedback regulation, we have revised the manuscript to point out that the PLT expression dynamics likely reflects not fully understood indirect feedback in the system. Indeed, also our own findings (Fig. 3A, see the response to point 2) points to feedback regulation from the post MZ onto the QC and early MZ. Please see also our response to Reviewer 4.

Minor comments

5. Fig. 1A and Fig. 2A. The UMAPs in Fig. 1A and Fig. 2A would benefit from clearer cell-type or tissue-region annotations. Since the downstream analyses rely heavily on the identification of SCN, QC, columella, cortex, and endodermis populations, direct labeling in the main figures would greatly improve readability.

We have added relevant labels to these figures.

6. Fig. 1E and the rationale for GATA4 inclusion later in the manuscript. Fig. 1E lists marker genes used to define different developmental phases. Since GATA4 is later analyzed as a late-MZ-associated SF1-GATA factor, the authors should explain why GATA2, but not GATA4, was included in Fig. 1E. If GATA4 is less restricted to the late MZ than GATA2, this should also be more clearly discussed when GATA4 is introduced as a late-MZ regulator.

GATA4 has now been added to Fig. 1E. We have also added a new panel to Fig. S2 illustrating, among other genes, the expression profiles of both *GATA2* and *GATA4* along the GT trajectory (Fig. S2E). The scRNA-seq data shows similar expression profiles for both genes (see also Fig. S3G). Although expression of *GATA4* appears to commence slightly earlier in the cortex trajectory (Fig. 1E), expression for both *GATA2* and *GATA4* peaks in the late MZ and both genes are among the very few markers for this region (see Dataset S1). Reporter lines likewise show overlapping expression in the late MZ (Fig. 3D,E). See also our response to point 9.

7. Fig. 2B. Some labels in Fig. 2B appear to be corrupted and should be corrected.

This has been corrected in the updated manuscript.

8. GATA2/GATA4 expression patterns should be shown before Fig. 3C. Before presenting the CellOracle perturbation predictions in Fig. 3C, it would be helpful to directly show the expression patterns of GATA2 and GATA4 in the relevant scRNA clusters or along the cortex trajectory, for example using FeaturePlots, trajectory expression plots, or module scores.

Expression data for *GATA2* and *GATA4* along the cortex trajectory can be found in Fig. S3G, as well as in the updated heatmap of Fig. 1E. Expression data in the GT trajectory is shown in the new Fig. S2E (see also our response to Reviewer 3, minor point 3), and *in planta* reporter expression for *GATA2* in Fig. 1M. All of these are referred to just before Fig. 3C is introduced in the updated manuscript.

9. The apparent inconsistency between GATA expression timing and predicted perturbation timing requires clarification. Based on the reporter images in Fig. 3D/E, GATA4 appears to initiate later and to extend more broadly into the post MZ, whereas GATA2 appears to initiate earlier and to be more spatially restricted. However, in the CellOracle perturbation analysis shown in Fig. 3C, the predicted effect of GATA4 loss appears to occur at an earlier developmental stage than that of GATA2. This is not immediately intuitive, because one might expect the perturbation timing to broadly correspond to the spatial-temporal expression

domains of the respective TFs. Additional evidence or robustness analysis would be helpful to support the reliability of the differential perturbation patterns predicted for GATA2 and GATA4.

Indeed, the outcomes of TF perturbation in CellOracle are connected to the spatial-temporal expression domains of the respective TFs. As mentioned in Minor Point 6, expression profiles for *GATA2* and *GATA4* along the cortex trajectory are similar, showing maximum expression in the late MZ, but expression of *GATA4* commences slightly earlier. With regards to reporter data, it is important to note that independent reporter lines can show slight changes in expression though the general pattern is consistent. Tens of independent transformants were analyzed for each reporter line. In all cases, as in the scRNA-seq, expression of both GATAs is strongest in the late MZ, however, across independent transformants, the onset of expression can shift by a few cells. This is true for both GATA2 and GATA4. Since scRNA-seq data is profiling many roots at once, the expression profiles for the two GATAs is a consequence of averaging expression across hundreds of roots and as such may be a more robust representation of the biology. Given the precise RNA-seq expression profile, prolonged reporter expression into the post MZ, which also varies from line to line, likely reflects a distinction in RNA versus protein stability, as reported for the PLT TFs (Galinha et al., 2007). We have clarified the expression data for *GATA2* and *GATA4* in the updated manuscript.

10. The GATA2 reporter construct should be labeled consistently in Fig. 1M and Fig. 3D
Fig. 1M and the left panel of Fig. 3D appear to show the same type of GATA2 promoter reporter construct, but the construct names are labeled differently. Fig. 1M is labeled as pGATA2::2xYPET, whereas Fig. 3D is labeled as pGATA2::2xYPET-NLS. If the same construct was used, the construct name and figure legend should be made consistent to avoid confusion.

Thank you, this has been corrected in the updated manuscript.

11. The earlier onset of GATA2 expression in the epidermis than in GT should be discussed
In Fig. 1M and Fig. 3D, GATA2 expression appears to initiate earlier in the epidermis than in the GT/cortex. Since the manuscript mainly focuses on the GT trajectory, the authors should clarify whether SF1-GATA factors are proposed to coordinate the proliferation-to-differentiation transition across multiple tissues, or whether their timing and function differ between the epidermis and GT. If the former is the case, the apparent difference in the onset of GATA expression between epidermis and GT raises the question of whether the MZ/EZ transition is asynchronous across different tissues. This point would be worth briefly discussing.

We now mention this in the updated discussion.

Reviewer #2 (Remarks to the Author):

The publication discusses the role of GATA transcription factors in coordinating developmental progression in plants. The paper is significant because it uses single-cell transcriptomic data to provide deep functional insights into the process.
There have been significant publications on the GATA transcription factor in recent years, including a paper by Kim (2024) in Nature. However, most publications to date have focused on the expression patterns of the GATA transcription factors. To my knowledge, the work discussed in this publication is novel and provides significant insight.

The methodology is sound, in that the initial in silico analysis is followed by promoter fusion analysis and transient expression using protoplast. These results are further confirmed by GATA2 knockout mutant phenotypes. The inclusion of the mutant analysis is a strong positive point for the paper. In addition, the use of mutants and p35S:GATA4-GR lines shows that the authors conducted a strong genetics-based analysis to confirm the initial in silico findings. The methodology is sufficient for reproducibility.

In conclusion, I believe that this manuscript presents a thorough analysis of a novel aspect of the GATA transcription factors and oragan development in general. I recommend it for publication.

We thank the reviewer for the kind comments.

Reviewer #3 (Remarks to the Author):

The manuscript by Denyer et al. investigates root developmental progression in the Arabidopsis root ground tissue. By integrating scRNA-seq and scATAC-seq datasets, the authors identified a major transcriptional switch occurring in the late meristematic zone that mediates the differentiation of endodermis and cortex cells. Further analyses pinpointed and experimentally validated GATA transcription factors as key regulators of this transcriptional transition. Overall, this study reveals an exciting mechanism that coordinates cell proliferation and differentiation during root development. However, several issues need to be addressed before the conclusions can be fully supported.

Major issues:

1. The study relies primarily on a single scRNA-seq dataset (Dataset S1, Fig. 1A) for downstream analyses. Since several additional Arabidopsis root scRNA-seq datasets have been published previously, it would be important to determine whether the identified developmental trajectories and transcriptional programs are reproducible across independent datasets. Additionally, because this study represents a re-analysis of a previously published dataset (Denyer et al., 2019), a brief description of the dataset, including the number of cells, sequencing platform, and major processing steps, should be provided. The marker genes used for cell cluster annotation should also be listed. Furthermore, Dataset S1 is not sufficiently self-explanatory and is difficult to interpret. Without access to the original Seurat RData objects, the information provided in the table is limited. Additional annotation information and explanations should be included to improve its usability.

We have re-formatted and added further detail and clarity to Dataset S1 with regards to marker genes and annotation of the clusters. Since it is the same dataset as in Denyer et al., 2019, albeit reprocessed, the same marker genes were used for annotation. For reprocessing, we used a newer CellRanger version and updated filtering parameters which have changed since single cell analysis has become more established. Reprocessing parameters are detailed in the Methods section. All Seurat objects, meta-

data and fragment files will be available to download from our online browser upon publication (www.zmbp-resources.uni-tuebingen.de/timmermans/plant-single-cell-browser/).

2. The authors define five expression phases along the developmental trajectory in Figure 1B and 1C: columella, QC, Early MZ, Late MZ, and Post MZ. This classification may be confusing because QC represents the starting point of both the columella lineage toward the root tip and the meristematic zone lineage toward the shootward direction. A clearer organization would be to define QC as the initial developmental stage, with columella representing one differentiation trajectory, while Early MZ, Late MZ, and Post MZ represent subsequent developmental phases (e.g., phases 2, 3, and 4) along the ground tissue lineage.

It is true that the trajectory is not a linear one and that it branches in two directions from the QC. While the columella cells are not part of the actual GT trajectory, we include them as this proved to greatly improve the trajectory's cellular resolution. This is evident in the heatmap (Fig. 1C) and the expression patterns of associated reporter lines (Fig. 1F-Q, Fig. S2D). In the updated manuscript we have pointed this out and have added arrows indicating the bidirectionality of the trajectory, from the QC, in Figures 1B and 1C.

3. GATA transcription factors constitute a large gene family, and functional redundancy among family members is likely. Therefore, the expression patterns of all GATA family members should be examined to determine whether GATA2 displays a unique expression pattern or whether other GATA genes show similar expression dynamics during root development.

The GATA TFs show a remarkable expression variety in the root (see Fig. S3G as an example). However, only *GATA2*, *GATA4*, and to a lesser extent, *GATA12*, show an expression peak in the late MZ. Importantly, CellOracle identified specifically GATA2 and GATA4 as key regulators that region of the root. This does not preclude additional redundancy or compensation from other GATA TFs (see also the response to point 7 below), and we have highlighted this more clearly in the updated text.

4. In Figure 2A and Figure S3C, the criteria used to select cells for trajectory analysis are unclear. Compared with the scRNA-seq UMAP, additional cell groups appear at the terminal branches of cortex and endodermis trajectories but were not included in the trajectory analysis. The authors should clarify how cells were selected and explain why these populations were excluded.

Equivalent nuclei to the cells of the scRNA-seq trajectory were selected from the scATAC-seq atlas, based on label transfer methods. Since ATAC-seq requires nuclei rather than cells, there is no cell size bias in that atlas, which is largely unavoidable in cell-based microfluidics-based methods (see also response to Reviewer 1, Point 2, Denyer and Timmermans, 2022). As such, large differentiated cells, while captured in the scATAC-seq atlas, are largely missing in the scRNA-seq equivalent. We have clarified this point in the reviewed manuscript.

5. In Lines 204–205, the authors state that "more DEGs proximal to post-MZ DARs are expressed preferentially in the QC and early MZ." This statement requires clarification. Does this indicate that the corresponding chromatin regions are inaccessible in QC and early MZ but become accessible in post-MZ cells? However, several of these genes, such as WOX5 and TMO7, are already expressed in QC or early MZ cells, which appears contradictory. The relationship between chromatin accessibility dynamics and gene expression patterns should therefore be clarified.

Post MZ DAR indeed refers to a genomic fragment that is inaccessible in the QC and early MZ but accessible in post MZ cells. However, a gene can be proximal to multiple accessible chromatin regions. When a given gene is, for example, proximal to DARs in both the QC and the post MZ, but is expressed specifically in the QC, the chromatin accessibility in the QC can be interpreted as permissive for transcription, whereas the region of accessibility in the post MZ would be indicative of active transcriptional repression, specifically there. The profiles we see in Figure 3A are indicative of a dual regulation, and fit with the proposed role of the GATAs. We have noted this in the revised manuscript.

6. In Figure 3B, although several motifs show increased activity during the late MZ stage, GATA1, GATA4, and GATA12 motifs are not among the most strongly enriched motifs, and their enrichment decreases in the post-MZ stage. How do the authors reconcile this transient and intermediate enrichment pattern with the proposed central role of GATA factors in controlling the developmental transition?

CellOracle predicted a prominent role for the SF1 GATA TFs in the late MZ where the switch in gene expression programs from proliferation to differentiation occurs. That the SF1 GATA DNA-binding motifs are not the most enriched motifs in this region nicely illustrates the value of the applied machine learning approach. Binding motifs for the SF1 GATA and GRF TFs are indeed uniquely enriched in the late-MZ DARs (motifs for other TF families show prominence in multiple regions). This matches the expression- and protein accumulation patterns of GATA2, GATA4 and GATA12 (Figs 1E, S2F, 3D-E, S3G), and points to these GATAs functioning largely in this position along the root. Enrichment of other motifs in the late MZ DARs indicates contributions from other TFs as well. Indeed, it is to be expected that genes are regulated by more than one class of TFs.

7. In Figure 4D, the authors examine MZ cell numbers in the gata2 gata4 double mutant. It would be important to discuss whether other GATA family members are still expressed in the MZ region and whether they could partially compensate for the loss of GATA2 and GATA4 functions.

GATA12 is one of the closest relatives to GATA2 and GATA4 and is likewise preferentially expressed in the late MZ (Reyes et al., 2004; Fig. S3G). We expect that GATA12 is to some degree redundant with GATA2 and GATA4, and such redundancy has been proposed previously (Jiang et al., 2017). Other GATA family members are also expressed in the late MZ, albeit less specifically, suggesting potential for additional redundancy or compensation mechanisms. We have indicated this more prominently in the updated manuscript.

8. In Lines 354–394, the authors describe an interaction network between GATA factors and auxin signaling components (PLTs) as well as cytokinin signaling components (ARRs). However, whether these interactions are functionally important for root development remains largely untested. Therefore, the section title, "GATA TFs govern developmental progressions in a network integrating auxin and cytokinin signaling," may overstate the conclusions. A more appropriate title reflecting the observed associations rather than direct functional integration would be preferable.

We are not quite sure we understand the reviewer correctly. Our data shows that the SF1 GATAs directly target PLT and ARR genes (Fig. 5A, S6, Dataset S4). Conversely, expression of *GATA2* and *GATA4* is responsive to short-term treatments with auxin and cytokinin, as well as induction of PLT and ARR (Figs 5B-D). GATA2 and GATA4 further regulate a range of genes acting in the early- or post-MZ (Figs 4A-C, Dataset S4). Further, *gata2 gata4* mutants show delayed differentiation, whereas overexpression of GATA4 induces premature differentiation (Figs. 4D-E).

Minor issues:

1. In Figure 1, it is unclear which clusters in the UMAP (Figure 1A) correspond to columella, QC, endodermis, and cortex populations. It would be helpful to provide a supplemental figure showing Figure 1B using the same color scheme as Figure 1A, allowing readers to easily understand which cell populations were selected for trajectory analysis.

We have updated Fig. 1A with labels (see also the response to Reviewer 1, point 5). Coloring Fig. 1B to reflect which clusters the cells came from originally may not be too informative as cells resolve differently in the overall atlas versus the trajectory. This is particularly evident for QC/early MZ cells which distinguish clearly in the trajectory, but less so in the large meristematic cluster (3) of the atlas. Figure S2A shows the cells selected for the trajectory and one can see in this UMAP the general trend of the trajectory already.

2. In Figure 1C, the criteria used to define QC, Early MZ, Late MZ, and Post MZ stages should be clarified. The y-axis of Figure 1C is also confusing. Does it represent groups or modules of marker genes associated with each developmental stage? Similarly, in Figures S2B and S2C, representative gene names should be provided to facilitate interpretation.

Genes were placed on the y axis of the heatmap in order of where their expression peaks along pseudotime. This revealed distinct waves of gene expression that could be annotated based on expression profiles of known marker genes and newly generated reporter lines, as well as outcomes from GO-term enrichment, Pearson's correlation, and cluster analyses. These analyses also allowed the gene expression phases to be placed in an anatomical context (QC, early MZ etc…, Figs 1F-Q, S2D, Dataset S1), and these divisions are shown on the heatmap. We have clarified this in the updated legend. We hope that the representative genes shown in Fig. 1E, and now in new Fig. S2E (see point

3, below), are sufficient for interpretation of Fig. 1C. The two supplemental heatmaps (Figs S2B, C) are intended to illustrate the presence of defined gene expression waves also along the ground tissue and endodermal-terminal trajectory, as well as the position of the branchpoint in the late MZ.

3. The expression patterns of selected marker genes, particularly those associated with branch points of the trajectory, should also be visualized on the UMAP shown in Figure 1B.

We have added a new panel to Fig. S2 with UMAPs showing expression of some of these key markers on the trajectory (new Fig. S2E).

4. In Figure 2B, the authors analyze 500–1000 bp selected regions together with 2.5 kb gene body regions. The rationale for selecting these regions is unclear. Why were promoter regions not included, considering that TF binding motifs are often located within promoter regions? Additionally, in Figure 3A, the criteria used to define "2.5 kb region-specific DARs" should be clearly explained.

The figure shows the cluster-aggregated chromatin accessibility in regions within 2.5kb of the gene body. This is in either direction of the respective gene and therefore includes the promoters. In fact, the accessible chromatin regions shown are largely in the promoters. We do not specify in Fig. 2B where the regions shown are positioned in each case (i.e. before, or after the gene body), since the purpose of the figure is to illustrate select examples of prominent differential accessibility used for annotation of the atlas. Additional information on differential accessible chromatin regions, including positions relative to gene bodies is provided in Dataset S3. We have clarified this in the figure legend.

5. In Lines 363–375, GATA factors appear to activate ARR expression while repressing PLT expression. The authors should discuss the dual regulatory nature of transcription factors, which can function as both activators and repressors depending on genomic context and interacting partners.

We have now stated this explicitly.

Reviewer #4 (Remarks to the Author):

The submitted manuscript investigates the role of GATA transcription factors (TF) during cell differentiation and fate acquisition along Arabidopsis root tip, using a mix of scRNA-seq, transgenic lines, protoplast assay and microscopy approaches.

The authors have exploited published scRNA-seq datasets, focusing on analysis of the cortex/endodermis cell fate split as the two cell lineage derive from a single common initial cell and appear well identified in the scRNA-seq datasets.

They generated lines expressing selected cell identity markers and lines co-expressing

confocal markers for cell identities and GATA expression to show the spatio-temporal sequence of genes expression in the root apex and in endodermis cells.

The authors also mapped chromatin accessibility and created an atlas of accessible chromatin regions, highlighting the overlap between clusters of chromatin accessibility profiles and clusters according to cell identities from scRNA-seq data. They focused on the dynamics of gene expression the cortical-terminal cells to identify the GATA TF family as potential master regulators for the identity acquisition in this lineage, through a combination of TF binding motifs analysis, gene network analysis and in silico modelling with CellOracle. The implication of GATA TF in the regulation of cell identity acquisition processes is further tested in vitro through protoplastic RNA-seq analysis and luciferase assay for putative GATA targets and in vivo through confocal observation of identity markers in gata double mutant background.

The study in itself is well built and very comprehensive and the results are impressive. I nevertheless have a few major questions and some minors remarks.

Major Reflexions :

- if the dynamics of gene expression are so similar in the two cell lineage considered (cortex/endodermis) until the very late MZ - what causes the switch to one or the other cell fate ? Is it linked with the fact that the endodermis layer is closer to the stele and receive additional positional information (hormone gradient ?) which would send it towards its specific fate compared to cortex ? This particular point warrant more discussion from the authors, especially so since they propose a model of progression through proliferation/differentiation regulated by the auxin/cytokinin balance, which is know to be implicated in radial patterning of the root as well and mechanisms such as lateral root organogenesis and patterning.

While early on the lineages are similar on a global transcriptomic level, they are not identical. Genes such as *AT1G62500* are uniquely expressed in the early cortex, whereas genes such as *SCARECROW* and *MIR166A* show expression specifically in the early endodermis. The action of transcription factors such as SCARECROW and SHORTROOT contributes to the timed distinction of the two lineages, and indeed, proximity of the endodermis to the vasculature plays a role (Petricka et al., 2012). However, our findings indicate that the expression signature associated with meristematic activity, including cell proliferation, overshadows the lineage-specific distinctions. As such, in the RNA-seq trajectory, cells derived from the early MZ cluster together. This point is clarified in the manuscript.

- in the gata2 gata4 mutant the MZ appear longer and holds more cells, indicating that the differentiation process seems to take longer than in WT - but it ultimately occurs despite the absence of GATA. This suggests an alternative way to regulate the cell differentiation, albeit slower than under GATA control, and the authors suggests potential GATA12 redundancy to explain the still completing differentiation course. Ideally, the phenotype of gata12 mutant and double/triple gata 2/4/12 should be investigated to corroborate the conclusion that the GATA family is the linchpin of the shift between proliferation and

differentiation. The definitive proof of this assertion would be for the differentiation to be blocked altogether, unable to progress in absentio of GATA TFs. Otherwise, the conclusion should be toned down.

Please see also the response to Reviewer 3, point 7. There is likely redundancy with other GATA family members, most obviously, although not exclusively, with GATA12, which shows a temporal expression pattern similar to GATA2 and GATA4 (Fig. S3G). In addition, given the nature of the PLT gradient, in which PLT levels are gradually depleted as cells divide and are displaced away from the PLT source at the niche, the transition from cell proliferation to differentiation may inevitably occur. The data provided support a role for SF1 GATA TFs regulating the shift between proliferation and differentiation (Figs 4D-F, 5A-D). Particularly the phenotype resulting from inducible GATA4 overexpression provides strong evidence that these GATA TFs play a key role in the transition and are sufficient to induce differentiation prematurely (Fig. 4D). However, we agree with the reviewer that it would be helpful to highlight the potential for redundancy within the GATA family, and have now done so in the revised manuscript.

Related to this, due to additional feedback in the system, the phenotypes of higher order *gata* mutants may not necessarily be more informative. As mentioned in the response to point 4 from Reviewer 1, *arr1arr10arr12* mutants show a collapsed root meristem phenotype, which is counterintuitive given that the ARRs promote cell elongation / differentiation. The *arr1arr12* phenotype is in this regard more informative (Dello Ioio et al., 2007; Argyros et al., 2008).

- The model presented in fig 5E is incoherent with the data from panel 5A : in the proposed negative feedback loop, there is no possibility for the observed resurgence of PLT expression. A potential compatible explanation would rather be that a gene downstream of GATA or ARR would instead promote the delayed PLT expression resurgence, providing retrocontrol on the whole regulatory cascade. The proposed model should include this to be coherent with the data supporting it.

Please see also our response to Reviewer 1, point 4. We have addressed this point in the revised manuscript. However, we opted to not change the model, as this focuses on regulatory interactions specifically between the GATA, PLT, and ARR TFs. That said, if so desired by the Reviewers and Editor, we could still change this.

Minor Questions & Remarks :

- Can the authors justify why they used the TAIR10 and 10.55 reference for their analysis, when the version 12 is available since February 2026 ?

Version 55 was released in 2022, and was commonly used at the time this study was initiated. To redo the analysis with the latest version would require an entire overhaul of all analyses, datasets, and figures, but with little upside. Considering the excellent correspondence between bioinformatic predictions and *in planta* validations throughout the manuscript, we have faith that the TAIR reference used is already of very high quality.

- L21 "where RNA…coordination" - please reformulate. While plants lack promoter-proximal pausing seen in metazoans, they exhibit other forms of Pol II regulation such as 3' Pol II pausing (e.g. https://doi.org/10.1038/s41467-019-12328-w), so 'not well established' is an overstatement.

3' Pol II pausing is a rather different phenomenon. Here proteins prevent run on transcription from an upstream gene into a gene located just downstream. The pausing we refer to is connected to "transcriptional priming", which allows the rapid and robust initiation of gene expression, for example in response to a change in developmental signals.

- L44 "Realizes" - unproper term, prefer the use of "induces"

This terminology has been updated.

- L46-47 "although…analogy" - Please remove. One can argue that the state of cell proliferation exists as an uphill part of the landscape, present as a transient state (i.e. a local minimum of altitude like a small indent in the landscape) of gene expression corresponding to a proliferating state, from which the cells can be pushed out to then go downhill to a true 'minimum' corresponding to a differentiated state.

This has been removed.

- fig 4 legend : (B) mention 'above' and 'below' subpanel image positionning - there is only left and right subpanel images ? please correct.

Thank you for pointing this out, this has been updated.

- figS2 legend : "progress left and rich" -> "left and right" / "top right is a heatmap (as in 1E) -> the heatmap is not in the top-right position.

Thank you for pointing this out, this has been updated.

- figS2, panel D : please precise the zones denomination (early/late MZ/post MZ) on the pictures.

This has been updated.

- figS2, panel D : could the authors provide complementary information on the CHERRY tagged protein stability, as while the expression of GATA2 appears transient in the provided heatmap, the expressed marked protein under pGATA2 is present in the cortex lineage past late MZ boundary as attested by RGI3 and AT1G62500 promoter activities ?

Please see our response to Reviewer 1, Point 9. That expression of the GATA2 reporter persists beyond the late MZ boundary (Figs 1M, S2D) likely reflects a distinction in RNA versus protein stability, as reported for the PLT TFs (Galinha et al., 2007). This is why we focus more on the onset of reporter expression when discussing expression profiles. In

addition, heatmaps emphasize expression maxima. As such, we would expect GATA expression to overlap somewhat with these targets into the Post MZ. We have clarified the expression data for *GATA2* and *GATA4* in the updated manuscript.

- minor typos : legends of multiple data sheets for supp dataset S1 "DEG were dientified" -> "identified"

- minor typos : supp dataset S3 legends for multiple data sheets "accesible regions" -> "accessible" / "cortex tajectory" -> "trajectory" / "change in accesibility" -> "accessibility" / "adjted p-value" -> "adjusted" / "cortex-terminal trajecotry" -> "trajectory" / "seperated by developemental region" -> "separated"

- minor typos : supp dataset S4 legends for multiple data sheets "seperated by developmental region" -> "separated" / "trasnient expression" -> "transient"

Thank you for pointing out these typos, they have been updated.

References

- Argyros, R. D., Mathews, D. E., Chiang, Y. H., Palmer, C. M., Thibault, D. M., Etheridge, N., Argyros, D. A., Mason, M. G., Kieber, J. J., & Schaller, G. E. (2008). Type B response regulators of Arabidopsis play key roles in cytokinin signaling and plant development. The Plant cell, 20(8), 2102–2116. https://doi.org/10.1105/tpc.108.059584

- Cao, Z. J., & Gao, G. (2022). Multi-omics single-cell data integration and regulatory inference with graph-linked embedding. Nature biotechnology, 40(10), 1458–1466. https://doi.org/10.1038/s41587-022-01284-4

- Dello Ioio, R., Linhares, F. S., Scacchi, E., Casamitjana-Martinez, E., Heidstra, R., Costantino, P., & Sabatini, S. (2007). Cytokinins determine Arabidopsis root-meristem size by controlling cell differentiation. Current biology : CB, 17(8), 678–682. https://doi.org/10.1016/j.cub.2007.02.047

- Denyer, T., & Timmermans, M. C. P. (2022). Crafting a blueprint for single-cell RNA sequencing. Trends Plant Sci., 27, 92–103. https://doi.org/10.1016/j.tplants.2021.08.016

- Dorrity, M. W., Alexandre, C. M., Hamm, M. O., Vigil, A. L., Fields, S., Queitsch, C., & Cuperus, J. T. (2021). The regulatory landscape of Arabidopsis thaliana roots at single-cell resolution. Nature communications, 12(1), 3334. https://doi.org/10.1038/s41467-021-23675-y

- Farmer, A., Thibivilliers, S., Ryu, K. H., Schiefelbein, J., & Libault, M. (2021). Single-nucleus RNA and ATAC sequencing reveals the impact of chromatin accessibility on gene expression in Arabidopsis roots at the single-cell level. Molecular plant, 14(3), 372–383. https://doi.org/10.1016/j.molp.2021.01.001

- Fu, S., Wang, S., Si, D., Li, G., Gao, Y., & Liu, Q. (2025). Benchmarking single-cell multi-modal data integrations. Nature methods, 22(11), 2437–2448. https://doi.org/10.1038/s41592-025-02737-9

- Galinha, C., Hofhuis, H., Luijten, M., Willemsen, V., Blilou, I., Heidstra, R., & Scheres, B. (2007). PLETHORA proteins as dose-dependent master regulators of Arabidopsis root development. Nature, 449, 1053–1057. https://doi.org/10.1038/nature06206

- Gharui, S., & Sengupta, D. (2025). Molecular Interactions of the Pioneer Transcription Factor GATA3 With DNA. Proteins, 93(2), 555–566. https://doi.org/10.1002/prot.26749

- Jiang, K., Yung, V., Chiba, T., & Feldman, L. J. (2018). Longitudinal patterning in roots: a GATA2-auxin interaction underlies and maintains the root transition domain. Planta, 247, 831–843. https://doi.org/10.1007/s00425-017-2831-4

- May, G., Soneji, S., Tipping, A. J., Teles, J., McGowan, S. J., Wu, M., Guo, Y., Fugazza, C., Brown, J., Karlsson, G., Pina, C., Olariu, V., Taylor, S., Tenen, D. G., Peterson, C., & Enver, T. (2013). Dynamic analysis of gene expression and genome-wide transcription factor binding during lineage specification of multipotent progenitors. Cell stem cell, 13(6), 754–768. https://doi.org/10.1016/j.stem.2013.09.003

- Reyes, J. C., Muro-Pastor, M. I., & Florencio, F. J. (2004). The GATA family of transcription factors in Arabidopsis and rice. Plant Physiol., 134, 1718–1732. https://doi.org/10.1104/pp.103.037788

- Sun, K., Liu, X., Xu, R., Liu, C., Meng, A., & Lan, X. (2024). Mapping the chromatin accessibility landscape of zebrafish embryogenesis at single-cell resolution by SPATAC-seq. Nature cell biology, 26(7), 1187–1199. https://doi.org/10.1038/s41556-024-01449-0

- Zhang, L., He, C., Lai, Y., Wang, Y., Kang, L., Liu, A., Lan, C., Su, H., Gao, Y., Li, Z., Yang, F., Li, Q., Mao, H., Chen, D., Chen, W., Kaufmann, K., & Yan, W. (2023). Asymmetric gene expression and cell-type-specific regulatory networks in the root of bread wheat revealed by single-cell multiomics analysis. Genome biology, 24(1), 65. https://doi.org/10.1186/s13059-023-02908-x

Point-by-point responses to the Reviewers' comments:

Reviewer #1 (Remarks to the Author):

All my concerns have been addressed or clarified. Therefore, I am happy to recommend it for publication in nat comms.

Thank you

Reviewer #3 (Remarks to the Author):

The authors have thoroughly addressed all prior concerns.

Thank you

Reviewer #4 (Remarks to the Author):

I thanks the authors for their responses to my comments and to the other reviewers'. The revised manuscript is improved and satisfactory. My main remaining concern is still the model in figure 5E which has not been updated or nuanced to be coherent with the data and should be in my personal opinion considering its importance in the manuscript.

I leave the final appreciation of this point to the editor, and have no further comments at that stage.

We did not change the model in figure 5E, as this illustrates specifically the gene regulatory interactions between the PLT, SF1 GATA, and ARR transcription factors. As we now understand it, the Reviewer would like us to illustrate that additional feedback regulation from the late and post meristem zones on the niche and early meristem exist either outside or downstream of this network. As this is just a minor point in the paper, we believe that such additional arrows would rather distract from the main points.
